# AIP1 and cofilin ensure a resistance to tissue tension and promote directional cell rearrangement

**DOI:** 10.1038/s41467-018-05605-7

**Published:** 2018-09-10

**Authors:** Keisuke Ikawa, Kaoru Sugimura

**Affiliations:** 1 Institute for Integrated Cell-Material Sciences (WPI-iCeMS), Kyoto University, Kyoto, 606-8501 Japan; 2 JST PRESTO, Tokyo, 102-0075 Japan

## Abstract

In order to understand how tissue mechanics shapes animal body, it is critical to clarify how cells respond to and resist tissue stress when undergoing morphogenetic processes, such as cell rearrangement. Here, we address the question in the *Drosophila* wing epithelium, where anisotropic tissue tension orients cell rearrangements. We found that anisotropic tissue tension localizes actin interacting protein 1 (AIP1), a cofactor of cofilin, on the remodeling junction via cooperative binding of cofilin to F-actin. AIP1 and cofilin promote actin turnover and locally regulate the Canoe-mediated linkage between actomyosin and the junction. This mechanism is essential for cells to resist the mechanical load imposed on the remodeling junction perpendicular to the direction of tissue stretching. Thus, the present study delineates how AIP1 and cofilin achieve an optimal balance between resistance to tissue tension and morphogenesis.

## Abstract (web summary)

Cells must resist or release tension/compression when proliferating and moving during development. Here the authors show that AIP1, together with cofilin, promotes local actin turnover to regulate linkage between actomyosin and cell junctions and resist mechanical load imposed by tissue stretching.

## Introduction

The global patterns of forces in a tissue (e.g., tissue tension/compression) control many aspects of development including cell proliferation, cell rearrangement, and cell polarity^1–10^. Such control relies on the ability of cells to sense the distribution of forces and tune morphogenetic signaling pathways in response to the mechanical inputs. Moreover, cells must resist or release tension/compression when deforming, proliferating, and moving during development^2,11–13^. While an understanding of molecular mechanisms for stress generation has evolved in the past decade, much less is known on how cells respond to and resist such stresses at the molecular level during morphogenesis.

The actin cytoskeleton is capable of sensing and resisting applied forces both at the network and filament levels^14,15^. For example, mechanical strain on the actin network alters the structure of filamin A, which crosslinks the orthogonal filaments, thus inhibiting the binding between filamin A and a downstream signaling molecule^16^. Single actin filaments decrease their helical pitch when mechanically relaxed, and such structural changes are amplified through positive feedback between F-actin twisting and cofilin binding^15,17–19^. The actin network increases its elasticity or reorients the stress direction to resist applied forces by changing filament dynamics and/or network architecture^14,20,21^. Whether and how these force-responsive properties of the actin cytoskeleton and actin-binding proteins (ABPs) are involved in the development of multi-cellular tissue is largely unknown.

During morphogenesis, cells change their relative positions along the tissue axis by remodeling cell contact surfaces. This process, called directional cell rearrangement, shapes a tissue and develops its multi-cellular pattern^22–25^. The *Drosophila* pupal wing epithelium provides an excellent model system to study the mechanism through which tissue tension controls directional cell rearrangement. Starting ~15 h after puparium formation (h APF), forces generated in the hinge stretch the wing along the proximal-distal (PD) axis (Supplementary Figure 1a-d)^6^. The resulting anisotropic tissue tension acts as a mechanical cue to specify the axis of cell rearrangement^6–8,26^. Wing cells relocalize myosin-II (myo-II) at the adherens junction (AJ) that runs along the PD axis (PD junction) to resist tissue tension, and the balance between extrinsic stretching force and intrinsic cell junction tension favors PD cell rearrangement, thereby accelerating relaxation into a hexagonal cell pattern (hereafter called hexagonal cell packing; Supplementary Figure 1c, d)^7^. This relaxation may be primarily driven through interface mechanics, consistent with the observation of shear-induced reconnection of interfaces and hexagonal lattice formation in foam, non-biological soft matter^27,28^. However, in biological tissues such as the wing epithelium, interface mechanics must be orchestrated with molecular regulators of cytoskeleton and cell adhesion (e.g., force-responsive ABPs) responsible for responding to and resisting tissue tension. Answering the question in the wing should provide a general mechanism of epithelial development, as all cell rearrangements are associated with sensation and resistance to forces from the surrounding cells.

Here, we show that actin regulation mediated through actin interacting protein 1 (AIP1) and cofilin is responsible for supporting tissue tension-driven cell rearrangement and hexagonal cell packing in the *Drosophila* pupal wing. AIP1 is evolutionarily conserved from yeast to humans. In vitro studies have shown that AIP1 binds cofilin and F-actin and promotes F-actin severing via cofilin^29–32^. In vivo, AIP1 and cofilin control F-actin disassembly and remodeling during development^33–38^. We show that AIP1 is localized on the remodeling anterior–posterior (AP) junctions of wing cells, and tissue stretch is necessary for the biased distribution of AIP1. Inhibition of actin turnover by AIP1 or cofilin loss-of-function (l-o-f) results in the detachment of myo-II from the AP junctions, which hampers the stabilization of newly formed PD junctions. Interestingly, the disorder of junctional actomyosin is rescued by releasing tissue tension. Together, our data illustrate that actin turnover ensures a resistance to anisotropic tissue tension and promotes directional cell rearrangement by reinforcing the structural stability of remodeling junctions. This proposed mechanism is likely to be relevant to development of other epithelial tissues in which tissue tension coordinates with morphogenetic signaling pathways in individual cells.

## Results

### AIP1 is localized on remodeling AP junctions

To investigate the molecular mechanisms through which cells respond to and resist tissue tension during directional cell rearrangements in the *Drosophila* wing, we screened candidate ABPs (Supplementary Methods). First, we examined the subcellular distribution of 19 ABPs at 24 h APF. ABPs demonstrating interesting localization patterns were assayed for l-o-f phenotypes. Since the polygonal distribution of cells is much easier to measure than the dynamics of cell rearrangement, we used the fraction of hexagonal cells at 32 h APF, when the hexagonal cell packing process involving directional cell rearrangement is nearly complete, as a proxy for cell rearrangement defects. As described below, screening identified AIP1, which promotes F-actin severing via cofilin^29–32^, as a potential key regulator of cell rearrangement in the wing.

**Fig. 1:**
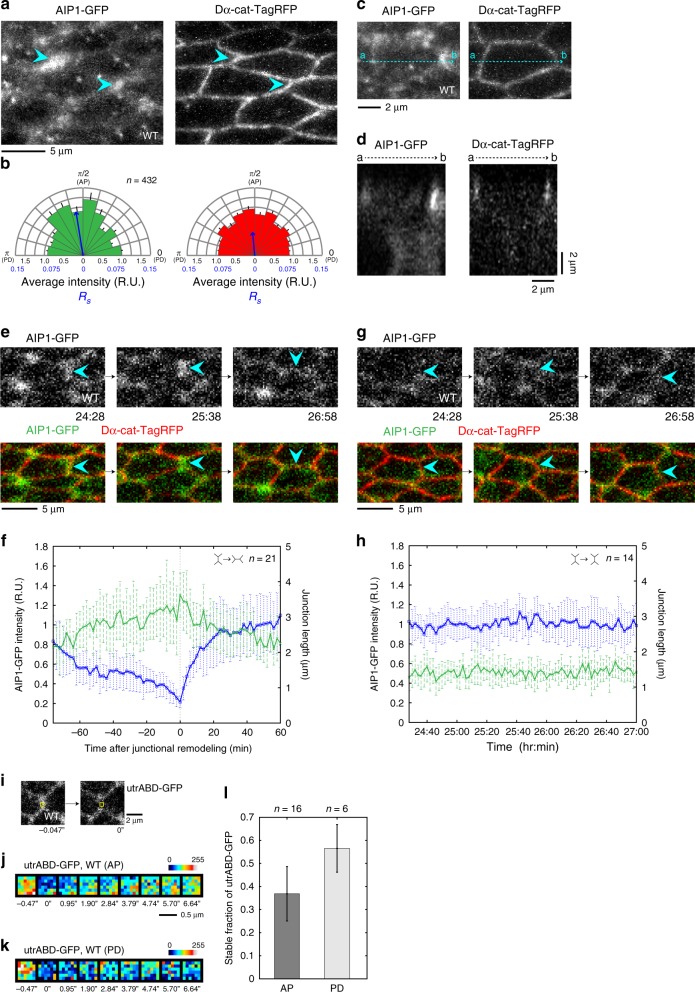
AIP1 localizes to remodeling AP junctions in the *Drosophila* pupal wing. **a** AIP1-GFP (left) and an AJ marker, Dα-cat-TagRFP (right) in the WT wing at 24 h APF. Blue arrowheads indicate AP junctions. **b** Directional bias of AIP1-GFP signal intensity (left) and Dα-cat-TagRFP signal intensity (right) along the junctions. Each junction is divided into twelve bins according to its angle relative to the PD axis, and the average signal intensity in each bin is plotted. Blue arrows indicate the length *R*_*s*_ and orientation *Θ *of an angular mean vector (Methods and Supplementary Figure 1e)^7^. **c**, **d** Top-view (**c**) and side-view (**d**) of cells expressing AIP1-GFP (left) and Dα-cat-TagRFP (right) at 24 h APF. The vertical section along the dashed arrow in panel **c** is shown in **d**. Alphabets indicate corresponding coordinates in different views. **e**, **g** Selected snapshots from movies of AIP1-GFP (gray in top panels, green in bottom panels) and Dα-cat-TagRFP (red in bottom panels). Blue arrowheads indicate remodeling (**e**) and stable (**g**) junctions. **f**, **h** Plot of AIP1-GFP signal intensity (green, left *y*-axis) and length (blue, right *y*-axis) of AP junctions undergoing cell rearrangement (**f**) or not (**h**). In **f**, *t* = 0 indicates the time point when the junction was reconnected. Data shown in **h** indicate that bleaching was negligible. **i** Snapshots of utrABD-GFP before and after photobleaching (square indicates ROI). *t* = 0 indicates the time point when photobleaching was conducted. **j, k** utrABD-GFP in ROIs examined before and after photobleaching along the AP (**j**) and PD (**k**) junctions. Color maps are shown in the upper right. **l** The stable fraction of utrABD-GFP. Welch’s *t*-test: AP vs. PD, *P* < 0.01. The number of junctions examined is indicated (**b**, **f**, **h**, **l**). Data are presented as the mean ± s.e.m. (**b**) and as the mean ± s.d. (**f**, **h**, **l**). Scale bars: 5 µm (**a**, **e**, **g**), 2 µm (**c**, **d**, **i**), and 0.5 µm (**j**)

**Fig. 2:**
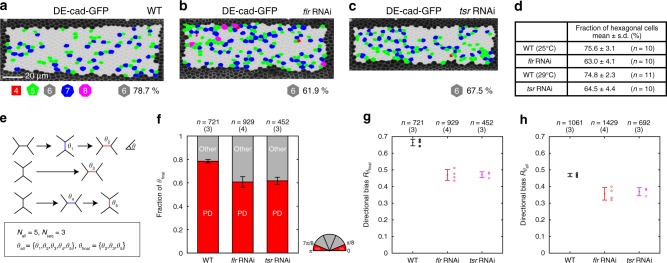
AIP1 and cofilin are required for the efficient hexagonal cell packing and directional cell rearrangement. **a**–**c** Images of DE-cad-GFP with the indicated genotypes (**a** WT at 32 h APF; **b** *flr* RNAi at 32 h APF; and **c** *tsr* RNAi at 28 h APF at 29 °C, which corresponds to ~32 h APF at 25 °C). Cells are colored according to the number of junctions (red, square; green, pentagon; gray, hexagon; blue, heptagon; and magenta, octagon). **d** Table of the fraction of hexagonal cells for each indicated genotype. Welch’s *t*-test: WT (25 °C) vs. *flr* RNAi, *P* < 0.001, WT (29 °C) vs. *tsr* RNAi, *P* < 0.001. **e** Schematic of cell rearrangement analysis. We tracked individual junctions that appeared in a movie and measured their angle relative to the PD axes (*θ*_i_) of newly generated junctions based on cell rearrangement. *θ*_final_ and *θ*_all_ are the sets of angles from the final round and all rounds of cell rearrangement, respectively, from which the magnitude of directional bias *R*_*θ*final_ and *R*_*θ*all_ were calculated (METHODS). *N*_seq_ and *N*_all_ indicate the numbers of remodeled junctions and cell rearrangements, respectively. **f**–**h** Quantification of cell rearrangement for each genotype based on time-lapse data captured at 24–27 h APF at 25 °C (WT, *flr* RNAi) and time-lapse data acquired at 20.5–23 h 20 m APF at 29 °C (*tsr* RNAi). **f** PD (red) and other (gray) fractions of *θ*_final_. Classification of *θ*_final_ is illustrated with a semicircle. **g**, **h** *R*_*θ*final_ (**g**) and *R*_*θ*all_ (**h**) of each genotype. **g** Dunnett’s test: WT vs. *flr* RNAi, *P* < 0.001, WT vs. *tsr* RNAi, *P* < 0.001. **h** Dunnett’s test: WT vs. *flr* RNAi, *P* < 0.01, WT vs. *tsr* RNAi, *P* < 0.01. The number of pupae (**d**), and the numbers of events and pupae examined (**f**–**h**) are indicated. Data are presented as the mean ± s.d. (**d**, **f**–**h**). Scale bar: 20 µm (**a**)

During the screening process, we detected a strong signal for GFP-tagged endogenous AIP1 along a subset of AP junctions at 24 h APF (Fig. 1a, b, Supplementary Figure 1e; see Supplementary Note 1 and Supplementary Figure 2a, c for characterization of *flare* (*flr*) (*Drosophila aip1* gene) protein trap line^36,39^). The directional bias in the AIP1 distribution became weaker at 28 h APF, when tissue tension is thought to contribute less to directional cell rearrangement (Supplementary Figure 1f, g)^7^. Time-lapse analysis confirmed that AIP1 accumulated on AP junctions during junctional remodeling, and AIP1 gradually disappeared from newly formed PD junctions (Fig. 1e,f; Supplementary Movie 1). In contrast, AIP1-GFP signal intensity remains low at stable AP junctions (Fig. 1g,h; Supplementary Movie 2), indicating that AIP1 specifically localizes to remodeling AP junctions. Fluorescence recovery after photobleaching (FRAP) of utrABD-GFP showed a smaller fraction of stable F-actin at the AP junctions than at the PD junction, which is in agreement with the AIP1 subcellular localization (Fig. 1i–l; Welch’s *t*-test, *P* < 0.01). Next, we examined the localization of AIP1 and its cofactor cofilin along the apico-basal axis. We confirmed that AIP1 is enriched at the AJ plane, whereas cofilin is diffusely distributed, as has been reported in the eye disc^36^ (Fig. 1c, d and Supplementary Figure 3f-h; see Supplementary Note 1 and Supplementary Figure 2b, c for characterization of *twinstar* (*tsr*) (*Drosophila cofilin* gene) protein trap line^40^). In the *flr* RNAi wing, hexagonal cell packing was disrupted, consistent with a defect in directional cell rearrangement (Fig. 2a, b, d; Welch’s *t*-test, *P* < 0.001). These results prompted us to further investigate a role for AIP1 in tissue tension-driven cell rearrangement.

### Tissue tension-dependent localization of AIP1

**Fig. 3:**
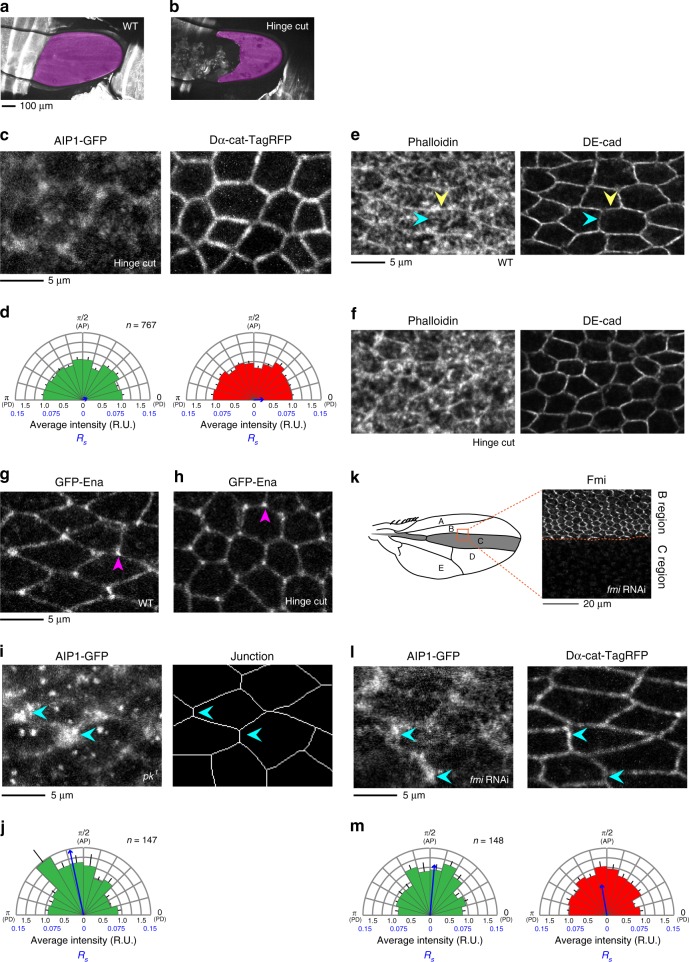
AIP1 localizes to AP junctions in a tissue stretch-dependent manner. **a**, **b** Low magnification images of DE-cad-GFP of the WT (**a**) and mechanically relaxed (**b**) wings at 24 h APF. The wing blade is shaded magenta. **c** AIP1-GFP (left) and Dα-cat-TagRFP (right) in the mechanically relaxed wing at 24 h APF. **d** Directional bias of AIP1-GFP signal intensity (left) and Dα-cat-TagRFP signal intensity (right) in the mechanically relaxed wing was quantified as described for Fig. 1b. **e**, **f** F-actin distribution in WT (**e**) and mechanically relaxed (**f**) wings at 24 h APF. Phalloidin (left) and DE-cad (right). Yellow and blue arrowheads indicate PD and AP junctions, respectively. **g**, **h** GFP-Ena in WT (**g**) and mechanically relaxed (**h**) wings at 24 h APF. Magenta arrowheads indicate the accumulation of GFP-Ena on cell vertices. **i** AIP1-GFP (left) in a *pk*^*1*^ wing at 24 h APF. Cell contours are depicted (right). Blue arrowheads indicate AP junctions. **j** Directional bias of AIP1-GFP signal intensity in a *pk*^*1*^ wing. **k** Left: Schematics of the wing. The expression domain of *ptc-Gal4* was shaded gray. A square indicates the region shown in right. Right: The Fmi antibody signal in a *fmi* RNAi wing at 24 h APF. **l** AIP1-GFP (left) and Dα-cat-TagRFP (right) in a *fmi* RNAi wing at 24 h APF. Blue arrowheads indicate AP junctions. The animal was carrying one copy of AIP1-GFP, and the brightness-contrast was adjusted using different parameters compared to those used in other homozygous samples. **m** Directional bias of AIP1-GFP signal intensity (left) and Dα-cat-TagRFP signal intensity (right) in a *fmi* RNAi wing. The number of junctions examined is indicated (**d**, **j**, **m**). Data are presented as the mean ± s.e.m. (**d**, **j**, **m**). Scale bars: 100 µm (**a**), 5 µm (**c**, **e**, **g**, **i**, **l**), and 20 µm (**k**)

To characterize the mechanism by which tissue tension acts on AIP1, we examined whether tissue tension is required to bias the subcellular distribution of AIP1. To this end, we relaxed the tissue stretch by detaching the wing from the hinge^6,7^. AIP1 was more evenly distributed along junctions in the mechanically relaxed wing (Fig. 3a–d). WT wing cells accumulate F-actin along the PD rather than AP junctions, and such F-actin localization becomes non-polarized by relaxing the tissue stretch (Fig. 3e, f). Therefore, our data argue against the possibility that the loss of accumulation of AIP1 along the AP junctions in Fig. 3c, d is simply a result of the change in F-actin localization. For comparison, the same experiment was performed with another ABP, Enabled (Ena), which promotes the elongation of actin filaments^41^. As shown in Fig. 3g, h, Ena retained its localization at junctions and vertices in the absence of an extrinsic stretching force. Thus, tissue tension-dependent subcellular localization is specific to a subset of ABPs, including AIP1 and myo-II^7,42^.

In addition to mechanical anisotropy, the wing develops a planar cell polarity (PCP) along the in-plane axis of the epithelium^43^, which may bias AIP1 localization. However, AIP1 localized at AP junctions following the l-o-f of two core PCP components, *prickle* (*pk*)^44^ and *flamingo*/*starry night* (*fmi*/*stan*)^45,46^ (Fig. 3i–m), indicating that PCP is not required for correct localization of AIP1.

### AIP1-cofilin are required for efficient cell rearrangement

Next, we examined whether and how the depletion of AIP1 affects directional cell rearrangement. We expressed dsRNA against *flr* using the Gal4-UAS system combined with a temperature shift (Supplementary Note 2). Under experimental conditions, GFP-tagged endogenous AIP1 was not detected in *flr* RNAi cells (Supplementary Figure 2d-f). We also confirmed that *flr* RNAi did not alter tissue stress anisotropy per se (Supplementary Figure 4; Dunnett’s test, *P* > 0.9)^47^. The angles of new junctions generated by cell rearrangement were measured in time-lapse movies obtained at 24–27 h APF. When junctions underwent multiple rounds of remodeling, final and preceding rounds of junctional remodeling were considered separately for analysis (*θ*_final_ and *θ*_all_, and *N*_seq_ and *N*_all_ in Fig. 2e; METHODS). This is because the former affects the steady alignment of hexagonal cells along the PD axis that accelerates the formation of a hexagonal pattern (Supplementary Figure 1d), whereas the latter is an intermediate process of searching for an efficient relaxation pathway^7,24^. *flr* RNAi did not lower the frequency of cell rearrangement (Supplementary Figure 5; Dunnett’s test, *P* > 0.05 for *N*_seq_/*N*_cell_ and *P* > 0.05 for *N*_all_/*N*_cell_). Both *θ*_final_ and *θ*_all_ became less biased toward the PD axis following *flr* RNAi, and the difference between WT and *flr* RNAi was larger for *θ*_final_ than for *θ*_all_ (Fig. 2f–h; Dunnett’s test, *P* < 0.001 for *R*_*θ*final_ and *P* < 0.01 for *R*_*θ*all_; semicircle in Fig. 2f shows angle classification). Contraction-elongation was affected by *flr* RNAi as expected from disoriented cell rearrangement (Supplementary Figure 6a, b).

As AIP1 is a cofactor of cofilin, we examined whether cofilin is involved in regulating AIP1 localization and PD cell rearrangement. In in vitro studies, the binding affinity of AIP1 for F-actin is much weaker in the absence of cofilin^30^, and cofilin binds to actin filaments for a longer period of time when F-actin severing is suppressed through the loss of cofactor proteins, including AIP1^31^. In *S. cerevisiae*, *cofilin* hypomorph mutation leads to the diffusive distribution of AIP1 in the cytosol, whereas *aip1* null mutation moderately alters the subcellular localization of cofilin^35^. We determined whether these phenomena were also observed in the *Drosophila* pupal wing. In *tsr* dsRNA-expressing cells, in which GFP-tagged endogenous cofilin fluorescence was not detected (Supplementary Figure 2j), AIP1 was diffusely distributed in the AJ plane, with little accumulation at the AJ plane along the apico-basal axis (Supplementary Figure 3b-e). *flr* dsRNA-expressing cells exhibited abnormal aggregates of cofilin in the cytosol (Supplementary Figure. 3f-k). The enrichment of cofilin at the junction is more evident along the plane of AJ, presumably because cofilin remained in actin cables along the AJ plane for a longer period of time. Collectively, AIP1 and cofilin are mutually dependent for their localization, and phenotype quality and strength were conserved between fly and yeast. Next, we addressed the requirement for cofilin in directional cell rearrangement. *tsr* RNAi, without changing the inferred tissue stress anisotropy (Supplementary Figure 4; Dunnett’s test, *P* > 0.9), caused defects in hexagonal cell packing (Fig. 2c, d; Welch’s *t*-test, *P* < 0.001), directional cell rearrangement (Fig. 2f–h; Dunnett’s test, *P* < 0.001 for *R*_*θ*final_ and *P* < 0.01 for *R*_*θ*all_), and contraction-elongation (Supplementary Figure 6c), similar to that caused by *flr* RNAi.

### The acute delocalization of AIP1 impairs cell rearrangement

**Fig. 4:**
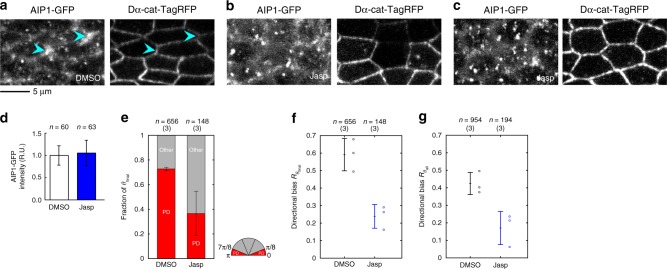
Jasp treatment results in disoriented cell rearrangement. **a**–**c** AIP1-GFP (left) and Dα-cat-TagRFP (right), in DMSO-treated (**a**) and Jasp-treated (**b**, **c**) wings at 24 h APF. Images in panels **b**, **c** show different regions of the same wing. Cells showing a faint signal of Dα-cat-TagRFP in **b** were located near the boundary of the *ptc-Gal4* expressing region. Blue arrowheads indicate AP junctions. **d** The average signal intensity of AIP1-GFP along junctions in DMSO- and Jasp-treated wings. Welch’s *t*-test: DMSO vs. Jasp, *P* > 0.2. **e**–**g** Cell rearrangement defects induced by Jasp treatment were quantified as shown in Fig. 2e–h. **e** PD (red) and other (gray) fractions of *θ*_final_. **f**, **g** *R*_*θ*final_ (**f**) and *R*_*θ*all_ (**g**) for each condition. **f** Welch’s *t*-test: DMSO vs. Jasp, *P* < 0.01. **g** Welch’s *t*-test: DMSO vs. Jasp, *P* < 0.05. The number of junctions (**d**), and the numbers of events and pupae examined (**e**–**g**) are indicated. Data are presented as the mean ± s.d. (**d**–**g**). Scale bar: 5 µm (**a**)

We also pharmacologically inhibited the functions of AIP1/cofilin using the F-actin stabilization drug Jasplakinolide (Jasp). Jasp stabilizes F-actin by competitively inhibiting cofilin binding to F-actin and decreasing the monomer off rate at filament ends^48,49^. As expected from the uniform distribution of AIP1 in *tsr* RNAi cells (Supplementary Figure 3c), the biased distribution of AIP1 was lost in wings incubated with Jasp for 4 h (Fig. 4a–c). In contrast, the average signal intensity of AIP1-GFP along junctions was not significantly affected (Fig. 4d; Welch’s *t*-test, *P* > 0.2). Under these conditions, the directionality of cell rearrangement was weakened (Fig. 4e–g; Welch’s *t*-test, *P* < 0.01 for *R*_*θ*final_ and *P* < 0.05 for *R*_*θ*all_), and the overall extent of contraction-elongation was decreased (Supplementary Figure 6d, e). In summary, the acute delocalization of AIP1 resulted in disoriented cell rearrangement, highlighting the importance of the subcellular localization of AIP1 in regulating directional cell rearrangement and suggesting that *flr* l-o-f phenotypes are unlikely to reflect early developmental defects.

Thus far, we showed that tissue tension specifies AIP1 localization in a cell, and AIP1 and its cofactor cofilin are required for efficient directional cell rearrangement. Thus, we next undertook experiments to answer three questions. First, how do AIP1 and cofilin promote directional cell rearrangement? Second, whether and how do AIP1 and cofilin facilitate a resistance to tissue tension during directional cell rearrangement? Third, how are AIP1 localization and function related to tissue tension?

### Fmi and Dsh polarities are largely normal in *flr* RNAi cells

Previous studies reported that hexagonal cell packing is regulated through the PCP pathway via cadherin trafficking in the *Drosophila* pupal wing, and AIP1 is required for PCP establishment^34,37,50^. Thus, AIP1 potentially controls cell rearrangement via the PCP pathway in the wing. However, we observed the enrichment of Fmi and Disheveled (Dsh)^51^ at AP (vertical) junctions in *flr* RNAi cells, similar to WT cells (Supplementary Figure 7b, d, f, h). To quantitatively evaluate these observations, we measured the orientation of Fmi and Dsh polarities in each cell using PCP nematics (Supplementary Figure 7a)^6^. According to this analysis, an off-axis fraction was detected for the Fmi polarity of *flr* RNAi cells (Supplementary Figure 7c, e; Watson’s test, *P* < 0.001), and Dsh polarity distribution was indistinguishable between WT and *flr* RNAi (Supplementary Figure 7g, i; Watson’s test, *P* > 0.4). The discrepancy with previous studies may result from different l-o-f conditions employed (see Supplementary Note 2 for details).

To address how the Fmi polarity map affects cell rearrangement, we transiently overexpressed *fmi-GFP*, which has been shown to be a functional allele^52^, and measured the orientation of cell rearrangement in such tissues. *fmi-GFP* overexpression abolished Fmi polarity (Supplementary Figure 8a, b; Watson’s test, *P* < 0.001), whereas the directionality of cell rearrangement was only mildly affected compared with that of *flr* RNAi cells (Supplementary Figure 8c-e; Welch’s *t*-test, *P* > 0.2 for *R*_*θ*final_ and *P* < 0.01 for *R*_*θ*all_). From these results, we concluded that cell rearrangement defects observed in the *flr* RNAi wing is unlikely to reflect PCP signaling dysfunction.

### The detachment of myosin cables along remodeling junctions

**Fig. 5:**
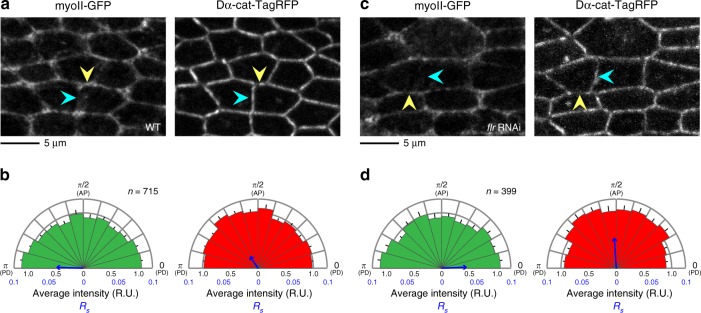
The global myo-II polarization pattern is not affected by *flr* RNAi. **a**, **c** myo-II-GFP (left) and the AJ marker Dα-cat-TagRFP (right) in WT (**a**) and *flr* RNAi (**c**) wings at 24 h APF. Yellow and blue arrowheads indicate PD and AP junctions, respectively. **b**, **d** Directional bias of myo-II-GFP signal intensity (left) and Dα-cat-TagRFP signal intensity (right) along junctions in WT (**b**) and *flr* RNAi **(d**) wings was quantified as have been done in Fig. 1b. The number of junctions examined (**b**, **d**) is indicated. Data are presented as the mean ± s.e.m. (**b**, **d**). Scale bars: 5 µm (**a**, **c**)

**Fig. 6:**
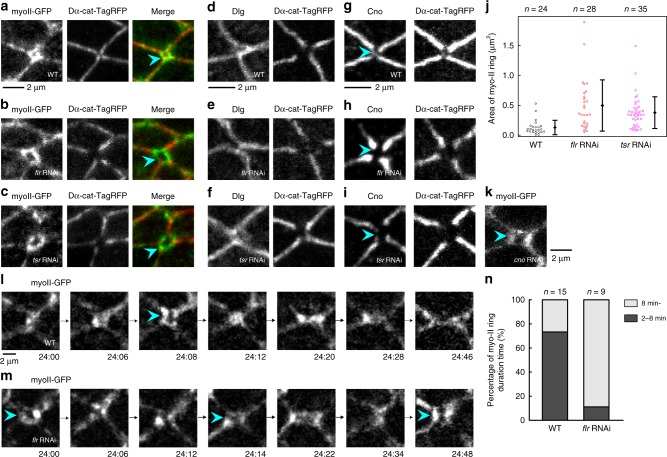
*flr* or *tsr* RNAi disrupts the Cno-mediated linkage between actomyosin and the AJ in remodeling junctions. **a** Images of myo-II-GFP (gray in a left panel, green in a right panel) and Dα-cat-TagRFP (gray in a middle panel, red in a right panel) in a WT wing at 24 h APF. The blue arrowhead indicates a “myo-II ring” along the short remodeling junction. **b**, **c** Images of myo-II-GFP (gray in left panels, green in right panels) and Dα-cat-TagRFP (gray in middle panels, red in right panels) in a *flr* RNAi wing at 24 h APF (**b**) and a *tsr* RNAi wing at 20.5 h APF at 29 °C, which corresponds to ~24 h APF at 25 °C (**c**). myo-II rings were enlarged in RNAi cells (blue arrowheads). **d**–**f** Images of a SJ marker, Dlg (left), and Dα-cat-TagRFP (right) in WT (**d**), *flr* RNAi (**e**), and *tsr* RNAi (**f**) wings. The stages examined are described in **a**–**c**. Dlg was present in short junctions where Dα-cat-TagRFP signals were faint. **g**–**i** Images of Cno (left) and Dα-cat-TagRFP (right) in WT (**g**), *flr* RNAi (**h**), and *tsr* RNAi (**i**) wings. The stages examined are described in **a**–**c**. *flr* or *tsr* RNAi resulted in the loss of Cno from short junctions (blue arrowheads). **j** The myo-II ring area is plotted for each genotype. Steel test: WT vs. *flr* RNAi, *P* < 0.001, WT vs. *tsr* RNAi, *P* < 0.001. The differences between WT and RNAi groups were statistically significant after excluding outliers above 1.2 µm^2^. **k** An image of myo-II-GFP in a *cno* RNAi wing at 24 h APF. *cno* RNAi induced the detachment of junctional actomyosin (blue arrowhead). **l**, **m** Time-lapse images of myo-II-GFP in WT (**l**) and *flr* RNAi (**m**) wings. Blue arrowheads indicate myo-II rings. The myo-II rings were not converted into stable PD junctions in *flr* RNAi cells. Note that GFP signal intensity was modified via image processing; thus, we only tracked structural changes in these images. **n** The fraction of myo-II ring time duration for each genotype. The number of ROIs examined (**j**), and the number of events examined (**n**) are indicated. Data are presented as the mean ± s.d. (**j**). Scale bars: 2 µm (**a**, **d**, **g**, **k**, **l**)

We next focused on myo-II, a well-established regulator of cytoskeletal dynamics during junctional remodeling^22–25^. Previously, we showed that myo-II is localized on PD junctions in response to tissue stretching^7^. This global myo-II polarization pattern was not affected by *flr* RNAi (Fig. 5). However, a local structural change in junctional actomyosin was observed during cell rearrangement. myo-II formed a small ring-like structure, accompanied by D-α-catenin (D-α-cat) signal loss, in the short junctions of WT cells (Fig. 6a; hereafter called the myo-II ring), and much larger myo-II rings were detected in *flr* or *tsr* RNAi cells (Fig. 6b, c, j; Steel test, WT vs. *flr* RNAi, *P* < 0.001, WT vs. *tsr* RNAi, *P* < 0.001). The septate junction (SJ) protein Discs large (Dlg) was present along short junctions (Fig. 6d–f), indicating that the myo-II ring structure did not represent cell extrusion; instead, it is generated by the detachment of myosin cables around the cell vertex (a similar subcellular structure was reported in ref.^53^), and the larger myo-II ring may represent its precocious formation. Time-lapse analysis showed that the myo-II ring was only transiently formed immediately prior to the reconnection of cell contact surfaces in WT cells. In contrast, the myo-II ring was present for a longer duration in *flr* RNAi cells and occasionally failed to convert into stable junctions, potentially resulting in disoriented cell rearrangement (Fig. 6l–n).

### Mechanical rescue of myo-II ring enlargement

**Fig. 7:**
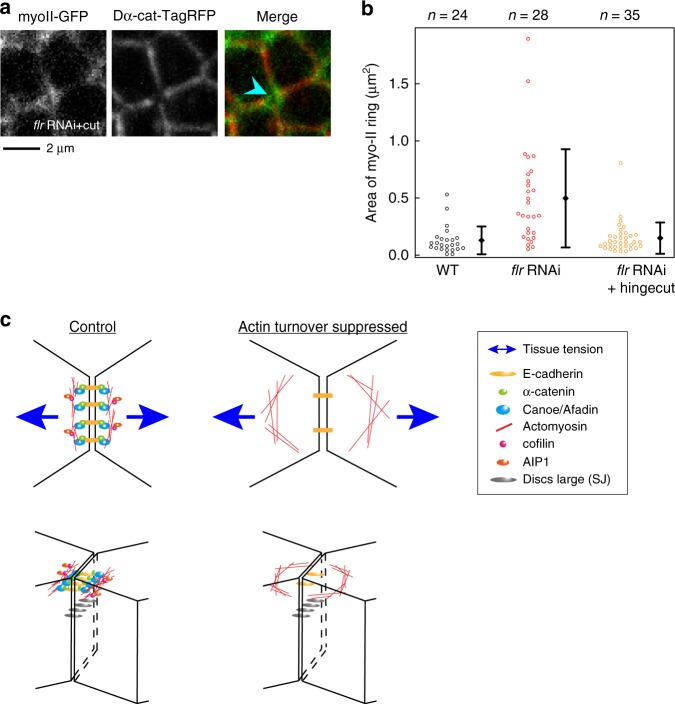
myo-II ring enlargement in *flr* RNAi cells is rescued by relaxing tissue stretch. **a** Images of myo-II-GFP (gray in a left panel, green in a right panel) and Dα-cat-TagRFP (gray in a middle panel, red in a right panel) in a mechanically relaxed *flr* RNAi wing. The wing was detached from the hinge at 15 h APF and observed at 24 h APF. The blue arrowhead indicates a myo-II ring on the short remodeling junction. **b** The myo-II ring area is plotted for each genotype (WT and *flr* RNAi data were same as that shown in Fig. 6j). Steel–Dwass test: WT vs. *flr* RNAi, *P* < 0.001, WT vs. *flr* RNAi + hinge cut, *P* > 0.4, *flr* RNAi vs. *flr* RNAi + hinge cut, *P* < 0.001. The difference between *flr* RNAi and *flr* RNAi + hinge cut was statistically significant after excluding outliers above 1.2 µm^2^. **c** Schematic of the defects resulting from genetic or pharmacological manipulations to stabilize F-actin. AJ (E-cad) (yellow), α-cat (green), Cno (light blue), actomyosin (red), cofilin (magenta), AIP1 (orange), Dlg (gray), and tissue tension generated by the extrinsic stretching force (blue arrows). Top (top) and side (bottom) views are shown. Left: In WT tissue, cells maintain AJ and actomyosin integrity along the AP junction in the presence of anisotropic tissue tension. Right: F-actin stabilization weakens Cno-mediated linkage between actomyosin and AJ. Subsequently, actomyosin detaches from the AP junction. The number of ROIs examined is indicated (**b**). Data are presented as the mean ± s.d. (**b**). Scale bar: 2 µm (**a**)

Interestingly, myo-II ring enlargement in *flr* RNAi cells was partially rescued by relaxing tissue stretch beginning at 15 h APF, when the extrinsic force begins to stretch the wing (Fig. 7a, b; Steel–Dwass test, WT vs. *flr* RNAi, *P* < 0.001, WT vs. *flr* RNAi + hinge cut, *P* > 0.4, *flr* RNAi vs. *flr* RNAi + hinge cut, *P* < 0.001). This result suggests that AIP1 prevents the detachment of junctional actomyosin, which runs perpendicular to the direction in which the wing is stretched, by anisotropic tissue tension (Fig. 7c).

### Actin turnover ensures mechanoresitance of junctions

To characterize the molecular mechanism by which AIP1 and cofilin regulate the reorganization of junctional actomyosin, we searched for molecules that connect actomyosin to the AJ in an AIP1/cofilin-dependent manner. Canoe (Cno) (*Drosophila* Afadin), which maintains the linkage between the actin cytoskeleton and the AJ in epithelial cells^12,53,54^, is one such candidate. We observed the specific loss of Cno in short junctions following *flr* or *tsr* RNAi (Fig. 6g–i). Moreover, *cno* RNAi induced the detachment of junctional actomyosin in wing cells (Fig. 6k). Together, these data indicate that disruption of the Cno-mediated linkage between actomyosin and the AJ leads to precocious formation of the myo-II ring.

**Fig. 8:**
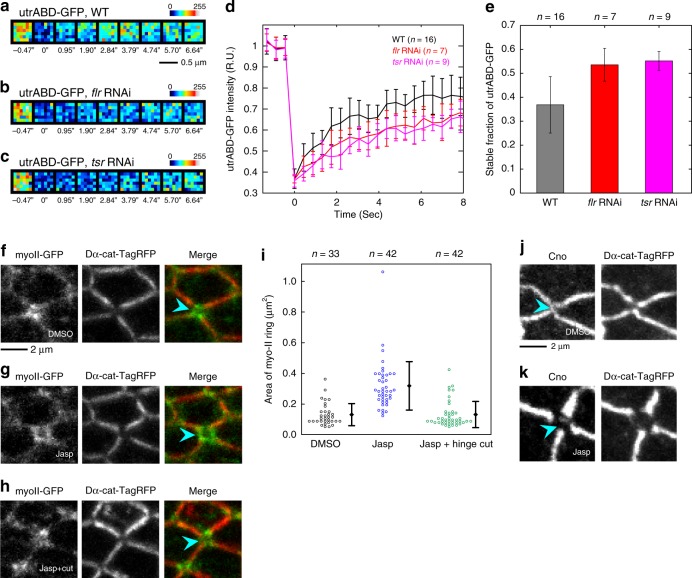
Actin turnover underlies the reorganization of actomyosin and cell adhesion. **a**–**c** utrABD-GFP in ROIs examined before and after photobleaching in the wings with the indicated genotypes (**a** WT at 24 h APF; **b**, *flr* RNAi at 24 h APF; and **c**, *tsr* RNAi at 20.5 h APF at 29 °C, which corresponds to ~24 h APF at 25 °C). Color maps are shown in the upper right. **d** Recovery of utrABD-GFP signal intensity after photobleaching at AP junctions (black, WT; red, *flr* RNAi; magenta, *tsr* RNAi). utrABD-GFP signal intensity was normalized by its average value at three time points prior to photobleaching. The average of the data from individual junctions is plotted. **e** The stable fraction of utrABD-GFP for each genotype. Steel test: WT vs. *flr* RNAi, *P* < 0.01, WT vs. *tsr* RNAi, *P* < 0.001. **f**–**h** Images of myo-II-GFP (gray in left panels, green in right panels) and Dα-cat-TagRFP (gray in middle panels, red in right panels) in a DMSO-treated wing (**f**), Jasp-treated wing (**g**), and Jasp-treated wing, which were detached from their hinges at 15 h APF (**h**). All images were acquired at 24 h APF. Blue arrowheads indicate myo-II rings. **i** The myo-II ring area is plotted for each condition. Steel–Dwass test: DMSO vs. Jasp, *P* < 0.001, DMSO vs. Jasp + hinge cut, *P* > 0.8, Jasp vs. Jasp + hinge cut, *P* < 0.001. **j**, **k** Images of Cno (left) and Dα-cat-TagRFP (right) in DMSO-treated (**j**) and Jasp-treated (**k**) wings at 24 h APF. Blue arrowheads indicate the short AP junctions. The number of junctions examined (**d**, **e**) and the number of ROI examined (**i**) are indicated. Data are presented as the mean ± s.d. (**d**, **e**, **i**). Scale bars: 0.5 µm (**a**) and 2 µm (**f**, **j**)

This observation is similar to that of a previous study in which the inhibition of actin turnover destabilized the attachment of the actomyosin network to AJs during apical cell constriction^55,56^. FRAP analysis consistently showed that AIP1 and cofilin were required for efficient actin turnover along AP junctions (Fig. 8a–e; Steel test, WT vs. *flr* RNAi, *P* < 0.01, WT vs. *tsr* RNAi, *P* < 0.001). In addition, ectopic F-actin accumulation, which may potentially reflect sites of impaired actin turnover, was detected in RNAi cells (Supplementary Figure 9). Application of the F-actin stabilization drug Jasp from 15 to 24 h APF induced myo-II ring enlargement and the loss of Cno from the short AP junctions (Fig. 8f, g, i–k). Furthermore, myo-II ring size returned to normal values following tissue stretch relaxation (Fig. 8h, i; Steel–Dwass test, DMSO vs. Jasp, *P* < 0.001, DMSO vs. Jasp + hinge cut, *P* > 0.8, Jasp vs. Jasp + hinge cut, *P* < 0.001). Collectively, these data show that AIP1 and cofilin promote actin turnover to regulate the Cno-dependent reorganization of junctional actomyosin in a specific region and at the appropriate time during cell rearrangement. This mechanism is essential for cells to resist the mechanical load imposed on the remodeling AP junction in a process of complete directional cell rearrangement.

### Cooperative cofilin binding links tissue tension and AIP1

**Fig. 9:**
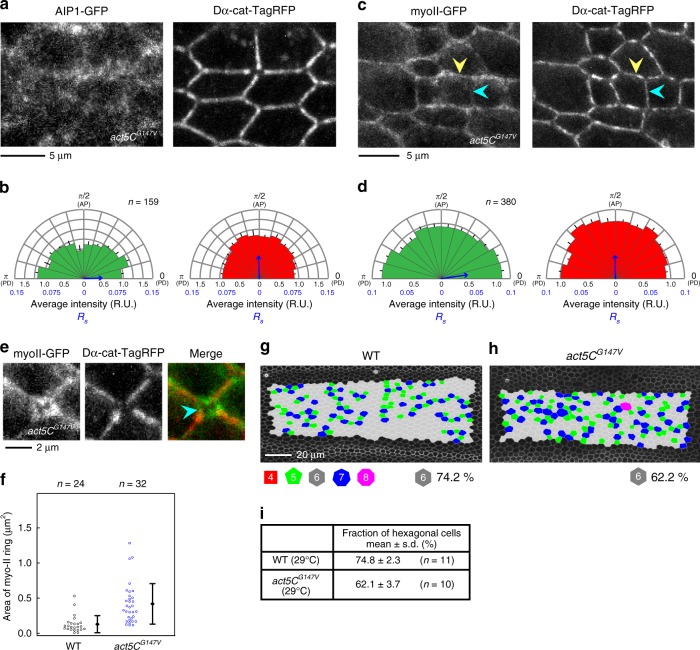
Cooperative binding of cofilin to F-actin is required for the biased distribution of AIP1, the integrity of junctional actomyosin, and hexagonal cell packing. **a** AIP1-GFP (left) and the AJ marker Dα-cat-TagRFP (right) in an *act5C*^*G147V*^ misexpressed wing at 20.5 h APF at 29 °C, which corresponds to ~24 h APF at 25 °C. **b** Directional bias of AIP1-GFP signal intensity (left) and Dα-cat-TagRFP signal intensity (right) along junctions in *act5C*^*G147V*^ misexpressed cells was quantified as described for Fig. 1b. **c** myo-II-GFP (left) and the AJ marker Dα-cat-TagRFP (right) in an *act5C*^*G147V*^ misexpressed wing at 20.5 h APF at 29 °C. Yellow and blue arrowheads indicate PD and AP junctions, respectively. **d** Directional bias of myo-II-GFP signal intensity (left) and Dα-cat-TagRFP signal intensity (right) along junctions in an *act5C*^*G147V*^ misexpressed wing. **e** Images of myo-II-GFP (gray in a left panel, green in a right panel) and Dα-cat-TagRFP (gray in a middle panel, red in a right panel) in an *act5C*^*G147V*^ misexpressed wing. Blue arrowhead indicates myo-II ring. **f** myo-II ring area. Wilcoxon rank sum test: WT vs. *act5C*^*G147V*^, *P* < 0.001. **g**, **h** Images of DE-cad-GFP with the indicated genotypes at 28 h APF at 29 °C, which corresponds to ~32 h APF at 25 °C (**g**, WT; h, *act5C*^*G147V*^ misexpression). Cells are colored according to the number of junctions. **i** Table listing the fractions of hexagonal cells for each genotype. Welch’s *t*-test: WT vs. *act5C*^*G147V*^, *P* < 0.001. The number of junctions examined (**b**, **d**), the number of ROI examined (**f**), and the number of pupae (**i**) are indicated. Data are presented as the mean ± s.e.m. (**b**, **d**) and as the mean ± s.d. (**f**, **i**). Scale bars: 5 µm (**a**, **c**), 2 µm (**e**), and 20 µm (**g**)

**Fig. 10:**
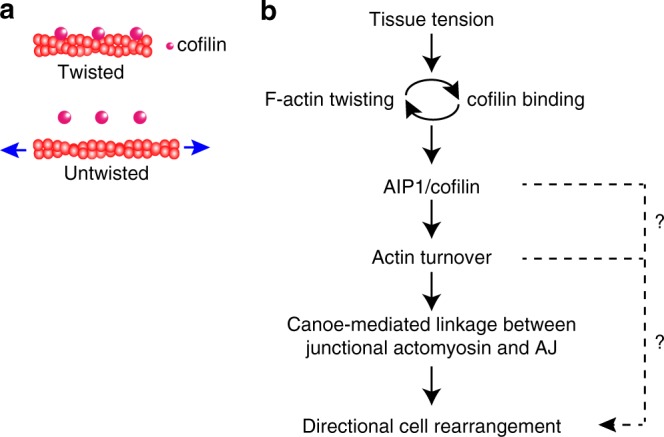
Summary and working hypothesis. **a** Schematic showing tension on actin filaments and their twisting, which have been reported by previous studies using in vitro reconstituted actin filaments^15, 17–19^. Actin filaments (red) alter their helical pitch in response to applied forces (blue arrows). cofilin (magenta) preferentially binds to twisted, mechanically relaxed actin filaments. **b** Summary of the working hypothesis. See the main text for details. Upper and bottom dashed arrows indicate an unidentified pathway regulating directional cell rearrangement downstream to AIP1/cofilin or actin turnover, which may potentially function via E-cad transport^36^

Finally, we addressed how tissue tension is related to AIP1 localization and function (Fig. 9). Actin filaments alter their helical pitch in response to applied forces, and these structural changes are amplified by positive feedback between cofilin binding and F-actin twisting (Fig. 10a)^15,17–19^. This suggests that in the pupal wing, F-actin along the PD axis, which is under tension, adopts a less twisted configuration, leading to the preferential binding of cofilin and AIP1 to more twisted F-actin along the AP axis via positive feedback between cofilin binding and F-actin twisting. To examine this hypothesis, we took advantage of an actin mutant that inhibits cooperative cofilin binding to F-actin (yeast *actin*^*G146V*^)^57^. Following misexpression of the corresponding mutant *Drosophila actin5C* (*act5C*) gene, AIP1-GFP did not accumulate at AP junctions (Fig. 9a, b). In contrast, the global polarity of myo-II distribution was not significantly affected, although myo-II-GFP signals became diffuse along the junction (Fig. 9c, d). Moreover, the misexpression of mutant *act5C* caused the detachment of junctional actomyosin and the AJ (Fig. 9e, f; Wilcoxon rank sum test, *P* < 0.001), resulting in impaired hexagonal cell packing (Fig. 9g–i; Welch’s *t*-test, *P* < 0.001). These developmental defects were specifically induced by the mutant Actin but not its wild-type counterpart (Supplementary Figure 10). Thus, our data support the hypothesis that the cooperative binding of cofilin to twisted, mechanically relaxed F-actin links anisotropic tissue tension and AIP1 localization and function (Fig. 10b).

## Discussion

In addition to global patterning determined by signaling molecules, global mechanical patterning represents another strategy for facilitating the long-range coordination of tissue development. This concept raises the questions as to how tissue-scale forces regulate biochemical signaling within or between cells and how cells subjected to tissue-scale forces maintain structural integrity when undergoing morphogenetic processes. The results of the present study highlight that actin regulation mediated through AIP1 and cofilin achieves an optimal balance between resistance to anisotropic tissue tension and morphogenesis.

The current working hypothesis (Fig. 10b) is that cells respond to anisotropy in tissue tension via the cooperative binding of cofilin to twisted F-actin, leading to AIP1 accumulation along remodeling AP junctions. AIP1 and cofilin facilitate a resistance to anisotropic tissue tension and maintain junction stability, thereby supporting PD cell rearrangement. Our observation that the misexpression of mutant *act5C* induced the delocalization of AIP1 and phenocopied *flr* RNAi is consistent with the current working hypothesis that postulates the positive feedback between cofilin binding and F-actin twisting. Future development of a biophysical method to visualize the helical pitch of actin filaments inside a cell will enable a more direct test.

We speculate that the myo-II ring is required to reconnect cell contact surfaces at four-way junctions. AIP1 and cofilin likely protect this temporally loosened junctional structure from anisotropic tissue tension by promoting actin turnover and strengthening the linkage between actomyosin and AJ via Cno, the *Drosophila* Afadin protein. Detailed molecular mechanisms that link actin turnover, junctional actomyosin, and E-cad/α-cat/Afadin remain elusive. Rapid actin turnover can prevent the aggregation of a contractile actin network and thereby maintain its uniform network structure^56,58^, which may be required for stable binding of F-actin to E-cad/α-cat/Afadin. AIP1-mediated actin turnover can also activate biochemical signaling that controls linker proteins, including α-cat and Afadin. In addition, it may potentially speed up lateral mobility of E-cad clusters to promote junctional remodeling^36,59^. Notably, candidate mechanisms mentioned above are not mutually exclusive. In the future, it would be necessary to address how different mechanisms act in concert to realize mechanoresistance of remodeling junctions.

This study uncovered a link between the biased distribution of AIP1 along the planar axis of a tissue and its function, a concept that was not addressed in previous studies investigating the role of AIP1 in tissue development^33,34,36–38^. Our data clearly indicate that tissue stretching is necessary for the AP-biased localization of AIP1; however, this observation also implies the existence of other factors because AIP1 accumulates specifically at remodeling AP junctions. Given that F-actin dynamically changes its distribution during development according to tissue tension patterns (Fig. 3e, f and our unpublished data), a more integrated understanding of actin dynamics and various ABPs is required to fully elucidate the regulatory mechanism underlying AIP1 localization.

In conclusion, the present study suggests two overlapping general strategies for the mechanical control of tissue development. First, tissue tension acts as a mechanical cue to bias protein localization and morphogenetic cell processes, thereby strengthening adjustability and flexibility to regulate development. The conversion of tissue tension into structural changes in the actin filament/network via ABP interactions may be a conserved strategy for mechanotransduction^60,61^, although conclusive, direct evidence in vivo is required. Second, global mechanical patterning must be reconciled to maintain the structural integrity of the cell/tissue. Future studies should explore whether the molecular mechanisms identified in the present study potentially function in other developmental contexts, such as cell proliferation and apoptosis, and/or in other epithelial tissues.

## Methods

### Generation of transgenic flies

To construct pUAST-*attB-act5C*^*WT*^, pUAST-*attB-act5C*^*G147V*^, and pUAST-*act5C*^*G147V*^, *act5C* was PCR-amplified from a cDNA clone (*Drosophila* Genomics Resource Center #LD18090). The G147V mutation was introduced using the GeneArt® Site-Directed Mutagenesis System (Thermo Fisher Scientific) with the following primer: 5′-GCTTTCTCTCTACGCCTCCGTCCGTACCACAGGTATCGTG-3′. Subsequently, *act5C*^*WT*^ or *act5C*^*G147V*^ was cloned into pUAST-*attB* or pUAST cut with EcoRI and NotI. The generated pUAST-*attB* and pUAST plasmids were injected into flies carrying 22A or 86Fa attP landing sites^62^, or *yw*, respectively, to produce transgenic flies.

### Inverse PCR

To determine an insertion site for the P-element containing an artificial exon encoding GFP in the *tsr-GFP* line (#ZCL0613)^40^, we followed protocols by BDGP (http://www.fruitfly.org/about/methods/inverse.pcr.html) and by Hoskins and Evans–Holm (http://flypush.imgen.bcm.tmc.edu/pscreen/files/GDP_iPCRProtocol_051611.pdf). Briefly, genomic DNA of the *tsr-GFP* line was extracted and subjected to restriction digestion using Sau3AI or HhaI, and the resultant DNA fragment was self-ligated. Then, the 3ʹ end of P-element and its flanking genomic DNA was amplified by using EY3.F and EY3.R primers for sequencing. The insertion site was determined using BLAST from FlyBase (http://flybase.org/).

### Western blotting

Pupae of *yw*, *flareGFP*, or *tsrGFP* at 24–28 h APF were lysed in Laemmili’s sample buffer (62.5 mM Tris (pH 6.8), 2% SDS, 10% glycerol, 0.001% bromophenol blue, and 713 mM β-mercaptoethanol). The lysates were boiled for 20 min and these samples were loaded into a 10% poly-acrylamide gel. After SDS-PAGE, samples were transferred to a PDMS membrane (Bio-Rad), blocked with 3% BSA-TBS (20 mM Tris (pH 7.2), 150 mM NaCl) and incubated with anti-GFP (1/1000, SantaCruz sc-9996) or anti-α-tubulin (1/2000, MBL PM054) diluted in 3% BSA-TBS overnight at 4 °C. The membrane was washed with TBS-0.05% tween20 and incubated with HRP-conjugated secondary antibodies (ThermoFisher) diluted in 3% BSA-TBS for at least 1 h at room temperature. Blots were developed using ECL Western Blotting Detection Reagents (GE healthcare) and imaged using LAS-4000 IR multi color (FUJIFILM). An original scan of the western blot is shown in Supplementary Figure 11.

### *Drosophila* genetics

Fly strains and a list of genotypes are summarized in Supplementary Methods.

### Immunohistochemistry and phalloidin staining

Anti-Cno (1/400)^63^, anti-Dlg (1/500)^64^, anti-Dsh (1/1000)^65^, and anti-Fmi (1/10)^45^ antibodies were used. Pupae at appropriate ages were dissected, and wings were fixed at room temperature for 30 min in PBS containing 4% paraformaldehyde. After washing with PBS containing 0.1% Triton X-100, these preparations were incubated overnight at 4 °C with the indicated antibodies.

To visualize F-actin in wing cells, dissected wings were incubated overnight at 4 °C with Alexa Fluor 546 Phalloidin (1/1000, invitrogen A22283). The condition of dissecting and fixing pupae was same as above.

### Image collection

To prepare the *Drosophila* pupal wing samples for image collection, pupae at appropriate ages were fixed to double-sided tape and the pupal case above the left wing was removed. The pupae were then placed on a small drop of water or Immersol W 2010 (Zeiss 444969-0000-000) in a glass bottom dish with the left side facing downward^6,7,46,66^. The fixed time-point images other than Fig. 3a, b and time-lapse images shown in Fig. 6l, m were acquired using an inverted confocal microscope (A1R; Nikon) equipped with a ×60/NA1.2 Plan Apochromat water-immersion objective at 25 °C. Other images were acquired using an inverted confocal spinning disk microscope (Olympus IX83 combined with Yokogawa CSU-W1) equipped with an iXon3 888 EMCCD camera (Andor), an Olympus ×60/NA1.2 SplanApo water-immersion objective, and a temperature control chamber (TOKAI HIT), using IQ 2.9.1 (Andor)^66^. After imaging, we confirmed that the pupae survived to at least the pharate stage.

### Surgical manipulation to relax the tissue stretch

Wings were detached from the hinges using forceps^6,7^. Hinges were severed at 23.5–24 h APF to analyze the effects of tissue tension on AIP1 localization (Fig. 3) or at 15 h APF for rescue experiments (Figs. 7, 8).

### Jasplakinolide treatment

A small drop of Jasplakinolide (Jasp) (Invitrogen J7473) solution, which was diluted to 100 µM or 200 μM in 10% or 20% dimethyl sulfoxide (DMSO; Nacalai #13408-64), was placed on a glass bottom dish. After removing the pupal case above the left wing, the pupae were placed in Jasp solution with the left side facing downward. Ten percent or 20% DMSO solution was used as the control. Filter papers soaked in DMSO or Jasp solution were placed along the pupae to prevent drying. For the acute treatment shown in Fig. 4, the pupae were incubated with 200 μM Jasp solution starting at 20 h APF and observed at 24 h APF. For the experiments shown in Fig. 8, the pupae were incubated with 100 μM Jasp solution starting at 15 h APF, when the extrinsic stretching force starts to act on the wing, and observed at 24 h APF. Note that previous study has required two to three orders of magnitude higher concentrations of drugs in experiments using the pupal wing, likely reflecting the fact that the drug solution must pass through the cuticle (Tadashi Uemura, personal communication)^46^. Therefore, the concentration of Jasp in wing cells was ~0.1–1 µM, which is within the range of values reported in other cells/tissues^48,49^.

### FRAP

*sqhp-utrABD-GFP* pupae at appropriate ages were mounted on an inverted confocal microscope (TCS SP8; Leica) equipped with a Leica HCX PL Apo ×63/NA1.2 water-immersion objective. To photobleach utrABD-GFP along the AP junction, the region of the interest (ROI; 0.54 μm × 0.54 μm) was irradiated for 0.1 s using a two-photon laser tuned to an 880-nm wavelength (Chameleon; Coherent). Images were collected at 0.437-sec intervals. Fluorescence intensities (FIs) of ROIs were measured using ImageJ. FIs were normalized as the average FI at three subsequent time points immediately prior to photobleaching and subtracted the residual value immediately after photobleaching. A FRAP recovery curve was constructed by fitting the normalized FI to FI(*t*) = *A*(1-exp(-*τ*^−1^*t*)) using MATLAB, from which the stable fraction of utrABD-GFP was calculated.

### Image analysis

#### Subcellular distribution of proteins

To characterize directional bias in the subcellular distribution of AIP1-GFP, myo-II-GFP, and the AJ marker Dα-cat-TagRFP, fluorescent signals along the AJ plane were extracted from a snapshot of live cells (0.094 µm/pixel). Subsequently, the following procedure was applied. First, the background signal was subtracted using the “subtract background” command (*r* = 100) in ImageJ. Second, to avoid counting the signals at the vertices for all associated junctions, we omitted the signals for 2 pixels at the ends of the junctions when calculating the mean signal intensity along each junction. Each junction was divided into twelve bins according to its angle relative to the PD axis, and the average signal intensity in each bin was plotted. In addition, we calculated the magnitude *R*_*s*_ and the orientation *Θs* of the polarity, defined as *R*_*s*_〈*s*〉e^*i*2*Θ*^ = 〈*s*e^*i*2*θ*^〉 − 〈*s*〉〈e^*i*2*θ*^〉 from a data set {*s*_*ij*_*,θ*_*ij*_}, where *s*_*ij*_ and *θ*_*ij*_ (0 ≤ *θ*_*ij*_ < *π*) are the fluorescent signal intensity and the angle of the contact surface between the *i*th and *j*th cells, respectively^7,47^. Larger values of *R*_*s*_ indicate the stronger bias toward a particular axis.

When analyzing time-lapse images (0.215 µm/pixel), we set *r* = 20 to subtract the background command. We omitted 1 pixel at the ends for junctions 6–8 pixels in length and 2 pixels at the ends for junctions longer than 8 pixels. The signal intensity along each junction was normalized to its temporal average.

In Fig. 3l, Fig. 9a, and Supplementary Figure 2e, f, the animal was carrying one copy of AIP1-GFP, and the brightness-contrast was adjusted using different parameters compared to those used for other homozygous samples.

#### PCP polarity

We followed a protocol described in a previous study^6^ to measure the orientation of Fmi, Fmi-GFP, Dsh, and DE-cad polarities. Briefly, each pixel in an ROI was grouped into bins according to its angle relative to a cell center (*N*_bin_ = 36; the size of bin is *π*/*N*_bin_), and the average signal intensity in each bin {*s*_*i*_*,θ*_*i*_} (*j* = 1, 2,..., *N*_bin_) was measured. Subsequently, the mean polarity angle *Θs* was calculated as cos 2Θ*s* $= Q_1{\mathrm{/}}\sqrt {Q_1^2 + Q_2^2}$, where $Q_1 = \mathop {\sum}\nolimits_{i = 1}^{N_{{\rm{bin}}}} {s_i\,\cos } \,2\theta _i$ and $Q_2 = \mathop {\sum}\nolimits_{i = 1}^{N_{{\rm{bin}}}} {s_i\,\sin } \,2\theta _i$, respectively.

#### Hexagonal cell packing

DE-cad-GFP images were skeletonized using ImageJ. From the skeletonized images, the position and connectivity of vertices were extracted, and the polygonal distribution of cells was measured in OpenCV. If the distance between the centers of the end pixels of an edge was smaller than 3 pixels in length, the edge was regarded as a four-way vertex^7^.

### Quantification of cell rearrangement process

Cell rearrangement was manually detected from time-lapse movies captured at 1-min intervals starting from 24 h APF at 25 °C (WT, *flr* RNAi, DMSO, Japs) or at 20 h 30 min at 29 °C (*tsr* RNAi). Subsequently, remodeling at each junction was automatically detected and manually assessed when necessary. By repeating this automatic detection and manual correction process, we estimated that >90% of cell rearrangement in a filmed region were counted in this analysis. *θ*_final_ and *θ*_all_ represent the sets of angles from the final round and all rounds of cell rearrangement, respectively. The magnitudes *R*_*θ*final_ and *R*_*θ*all_ (one minus circular variance) and the mean orientation of *θ* are given as *R*_*θ*_e^*i*2*Θ*^ = 〈e^*i*2*θ*^〉 (0 ≤ *θ*_*j*_ < *π*). Larger values of *R*_*θ*_ indicate the stronger directional bias. *N*_seq_ and *N*_all_ indicate the numbers of remodeled junctions and cell rearrangements in each movie, respectively. The frequency of cell rearrangement was calculated by dividing *N*_seq_ and *N*_all_ by *N*_cell_, which is defined as the average value of the number of cells at the first and last time points of a movie.

### Stress inference

Bayesian force/stress inference solves an inverse problem between forces (i.e., cell junction tension and cell pressure) and epithelial cell shape (i.e., position and connectivity of vertices that appear in an image)^47^ and provides relative values for cell junction tensions and cell pressures, allowing the global stress tensor to be calculated by integrating tensions and pressures^47,67^. The accuracy and robustness of force/stress inference have been rigorously tested both in silico and in vivo^47,68,69^. In this study, we quantified the normal stress difference *σ*_A_ ≡ (*σ*_*xx*_ – *σ*_*yy*_)/2 as a measure for the anisotropy of tissue stress from images of DE-cad-GFP.

### Statistics

Data are presented as the mean ± s.e.m. in plots of the signal intensity of AIP1-GFP, myo-II-GFP, and Dα-cat-TagRFP in each angular bin and as the mean ± s.d. in the other plots. *P*-values were calculated in R based on Welch’s *t*-test (Figs. 1l, 2d, 4d, f, g, 9i, Supplementary Figure 5c, d, Supplementary Figure 8d, e), the Wilcoxon rank sum test (Fig. 9f), Dunnett’s test (Fig. 2g, h, Supplementary Figure 4, Supplementary Figure 5a, b), the Steel test (Fig. 6j, Fig. 8e), the Steel–Dwass test (Figs. 7b, 8i, Supplementary Figure 10c, f), and a randomized version of Watson’s test (Supplementary Figure 7c, e, g, i, Supplementary Figure 8b). Parametric tests were selected when the number of samples in each group was less than six and/or data follow the normal distribution. The Dunnett or Steel test was performed when analyzing multiple comparisons between the WT and other groups (the Steel test is a non-parametric equivalent of Dunnett’s test). The Steel–Dwass test was performed when comparing every pair in all groups.

### Data availability

The authors declare that the data supporting the findings of this study are available within the paper and its Supplementary files, and available from the corresponding author upon reasonable request. Plasmids and fly stocks generated in this study are available from the corresponding author.

## Electronic supplementary material

Supplementary Information

Description of Additional Supplementary Files

Supplementary Movie 1

Supplementary Movie 2
