# Supplementary material for: AIP1 and cofilin ensure a resistance to tissue tension and promote directional cell rearrangement
Source: Nat Commun. 2018 Sep 10;9:3295. doi: 10.1038/s41467-018-05605-7 (PMC6131156; doi:10.1038/s41467-018-05605-7)
Supplement: Supplementary file 1 — Supplementary Information [file 41467_2018_5605_MOESM1_ESM.pdf]

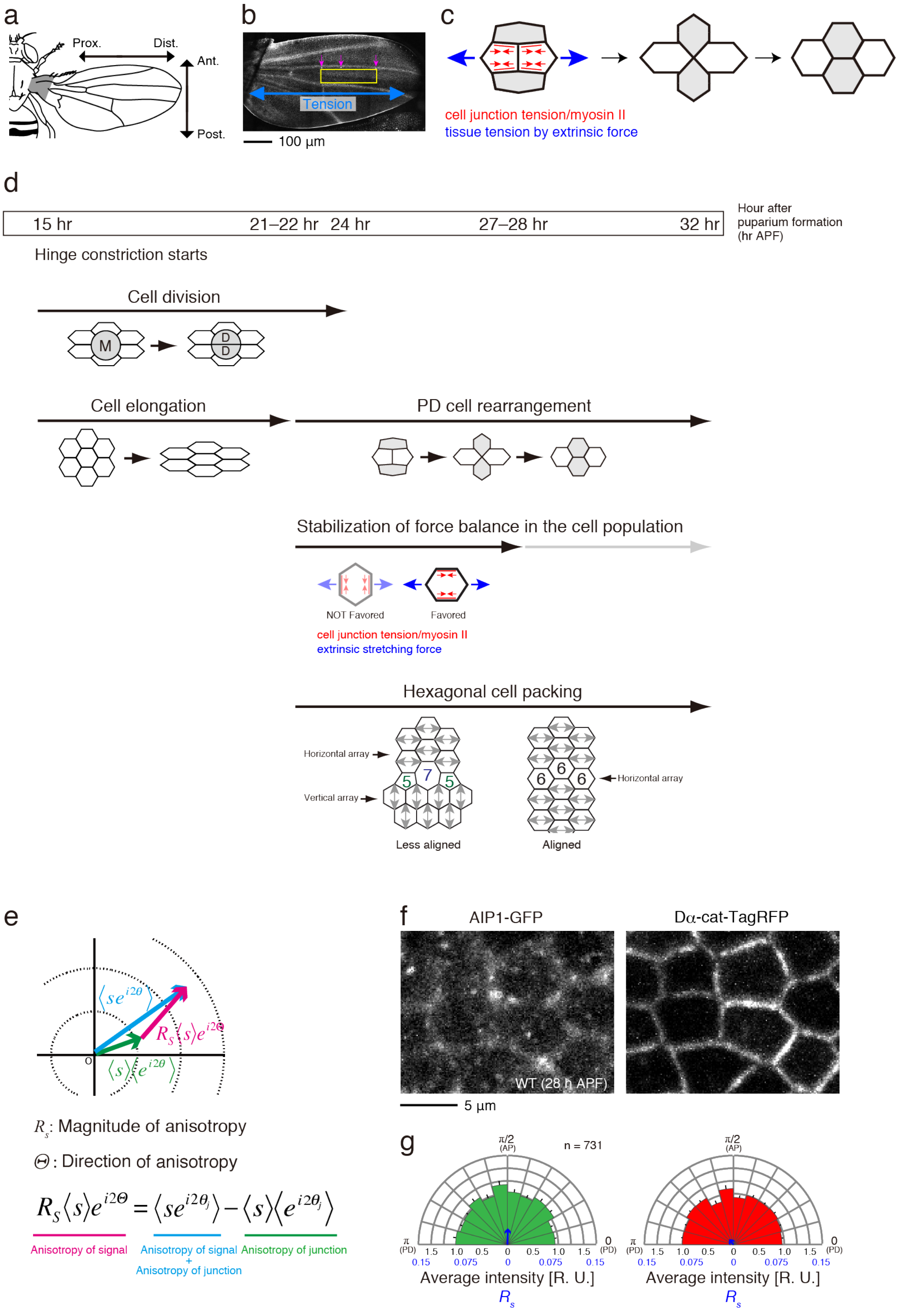

SUPPLEMENTARY FIGURE 1 Time course of the *Drosophila* pupal wing development.
(a) Schematics of the adult fly. The hinge is shaded gray. In this and all subsequent figures, the vertical and horizontal directions are aligned with the AP and PD axes, respectively.
(b) A wide image of the pupal wing at 24 h APF. Magenta arrows indicate 3 sensory organs on the L3 vein (L3-1 to L3-3). We set a line connecting L3-1 and L3-3 as the PD axis. We analyzed the intervein region between L3-1 and L3-3 (rectangle). The forces generated in the proximal region of the body stretch the wing along the PD axis (blue two-way arrow)[S01].
(c) Mechanical force balance triggers PD cell rearrangement in the *Drosophila* pupal wing. The intrinsic cell junction tension (red arrows) generated by myo-II (red lines) resists extrinsic stretching forces (blue arrows).

(d) Time course of *Drosophila* pupal wing development is illustrated based on the results of previous studies (adapted from ref. S02)[S01-S05]. Constriction of the hinge starts at 15 h APF, which leads to tissue tension along the PD axis in the wing. Upon tissue stretching, wing cells first elongate along the PD axis, and then the cells intercalate along the PD axis at 21-22 h APF and afterwards and adopt a more isotropic cell shape. The cells also proliferate until 24 h APF. The balance between intrinsic cell junction tension and the extrinsic stretching force favors PD cell rearrangement[S02]. The PD cell alignment promotes hexagonal cell packing by suppressing a mismatch between the orientation of hexagonal cell arrays[S02]. Thus, we conducted our analysis of cell rearrangement at 24–27 h APF when the tissue tension plays an important role and cell divisions mostly cease.
(e) Schematics of the anisotropy of subcellular distribution of proteins (adapted from ref. S02). The anisotropy of the signal intensity is represented by R_S, $R_S\langle s\rangle e^{i2\Theta} = \langle se^{i2\theta}\rangle - \langle s\rangle\langle e^{i2\theta}\rangle$, where s_{ij} and θ_{ij} ($0 \le \theta_{\mathrm{ij}} < \pi$) are the signal intensity and the angle of the junction between the *i*th and *j*th cells, respectively. R_S decreases if the signal intensity is uncorrelated with respect to the orientation of the junctions.
(f) AIP1-GFP (left) and the AJ marker, Dα-cat-TagRFP (right) in a WT wing at 28 h APF.
(g) Directional bias of the AIP1-GFP signal intensity (left) and the Dα-cat-TagRFP signal intensity (right) along the junctions at 28 h APF was quantified as described for Fig. 1b.
The number of junctions examined is indicated (g). Data are presented as the mean ± s.e.m. (g).
Genotypes are *DE-cad-GFP* (b), and *ptc-Gal4*, *UAS-Dα-cat-TagRFP*/+; *flr-GFP* (f, g).
Scale bars: 100 μm (b) and 5 μm (f).

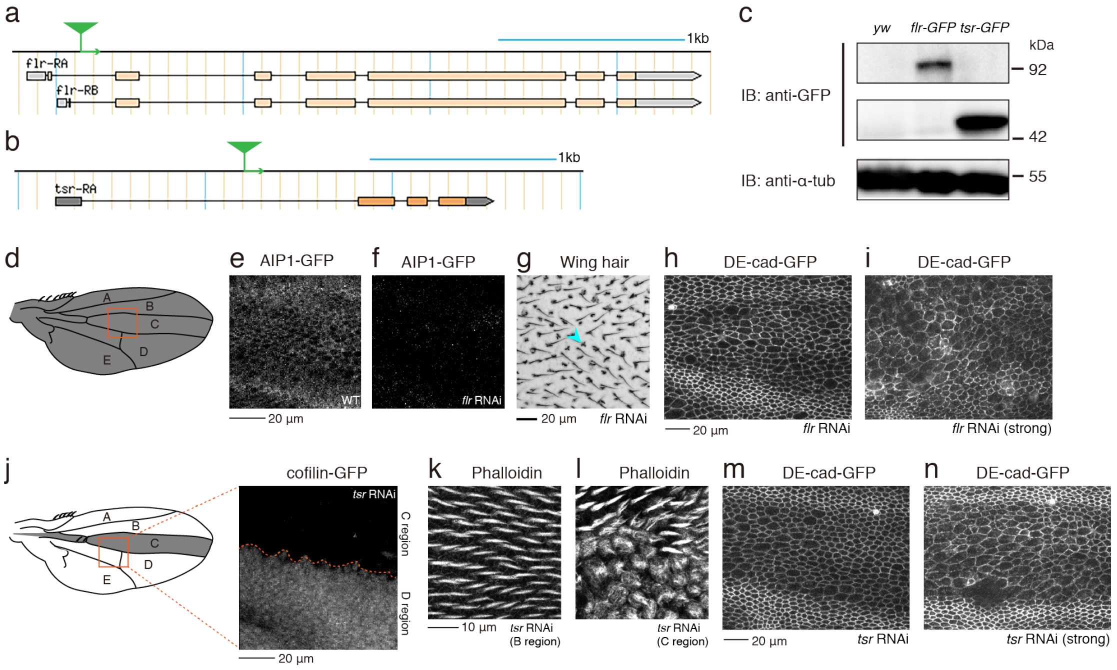

SUPPLEMENTARY FIGURE 2 Characterization of genetic tools used in this study.
(a, b) Schematic of *flr* transcripts (*flr*-RA and *flr*-RB) and the *flr-GFP* insertion site (a) and that of the *tsr* transcript (*tsr*-RA) and the *tsr-GFP* insertion site (b), which have been modified from Flybase (http://flybase.org/). Green triangles indicate the P-element insertion site. Orange and gray regions indicate the ORF and UTRs, respectively.
(c) Extracts of indicated genotypes were immunoblotted with anti-GFP (top and middle) or anti-α-tubulin antibodies (bottom). AIP1-GFP and Cofilin-GFP were detected at the expected molecular weight[S06]. Immunoblotting of α-tubulin was performed as a loading control. An original scan of the western blot is shown in Supplementary Figure 11.
(d) Schematics of the wing. The expression domain of *MS1096-Gal4* was shaded gray. The inset indicates the region shown in (e) and (f).
(e, f) AIP1-GFP in WT (e) and *flr* RNAi (f) wings at 24 h APF.
(g) An image of adult wing hairs of *flr* RNAi wing. Both normal and malformed (blue arrowhead) wing hairs were observed.
(h, i) Dependence of *flr* RNAi phenotype strength on timing of temperature shift. Images of DE-cad-GFP in the C region of *flr* RNAi wings at 24 h APF. In (i), temperature was shifted up at 25°C, 25 h earlier than (h) and other *flr* RNAi samples in this study.
(j) Left: Schematics of the wing. The expression domain of *ptc-Gal4* was shaded gray. The inset indicates the region shown on the right. Right: An image of Cofilin-GFP in a *tsr* RNAi wing at 20.5 h APF at 29°C, which corresponds to ~24 h APF at 25°C.
(k, l) Images of wing hairs of *tsr* RNAi wing at 31 h APF at 29°C, which corresponds to ~35 h APF at 25°C. Images in panels (k) and (l) show different regions of the same wing. Images in (k) and (l) show cells outside and inside the *ptc-Gal4*-expressing regions, respectively.
(m, n) Dependence of *tsr* RNAi phenotype strength on timing of temperature shift. Images of DE-cad-GFP in the C region of *tsr* RNAi wings at 20.5 h APF at 29°C. In (n), temperature was shifted up at 29°C, 3-5 h earlier than (m) and other *tsr* RNAi samples in this study.
Genotypes are *yw* (c), *flr-GFP* (c), *tsr-GFP* (c), *MS1096*; *UAS-Dα-catenin-TagRFP/+*; *flr-GFP/+* (e), *MS1096*; *UAS-Dα-catenin-TagRFP/UAS-flare dsRNA*; *flr-GFP/+* (f), *MS1096/X* or *Y*; *DE-cad-GFP/DE-cad-GFP*, *UAS-flr dsRNA* (g-i), *ptc-Gal4, tub-Gal80^{ts}, UAS-Dα-catenin-TagRFP/tsr-GFP, UAS-tsr dsRNA* (j), *ptc-Gal4*, *tub-Gal80^{ts}*, *DE-cad-GFP/DE-cad-GFP*, *UAS-tsr dsRNA* (k-n). Scale bars: 20 µm (e, g, h, j, m) and 10 µm (k).

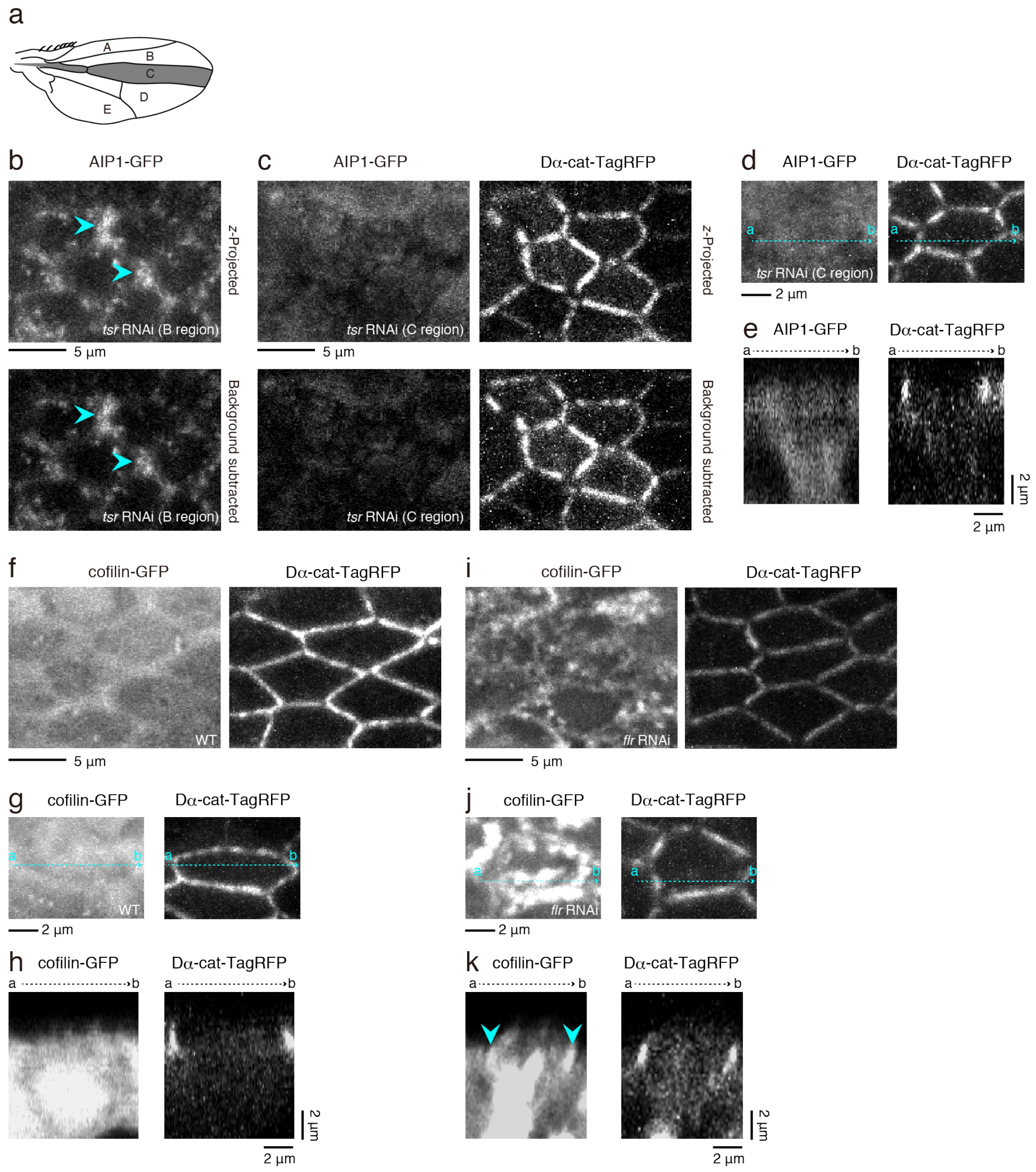

SUPPLEMENTARY FIGURE 3 AIP1 and cofilin are co-dependent in their localization.
In vitro studies have shown that the binding affinity of AIP1 for actin is much weaker in the absence of cofilin[S07]. To monitor this effect in live cells, budding yeast represents an excellent model system. AIP1 and cofilin specifically accumulate at a subcellular structure called the actin-patch, which is a region of the endocytic machinery[S08]. In a cofilin hypomorph mutant yeast cell, AIP1 is diffusely distributed. However, in an AIP1 mutant yeast cell, cofilin is recruited to actin cables as well as actin patches. Thus, AIP1 and cofilin demonstrate co-dependence for their subcellular localization. We examined whether this is also true in the *Drosophila* pupal wing.
(a) Schematics of the wing. The *ptc-Gal4*-expressing region is shaded gray. *ptc-Gal4* was used to drive the expression of Dα-cat-TagRFP as well as dsRNA against *flr* and *tsr*. Regions where data were obtained are indicated in corresponding panels.
(b-e) Images of *tsr* RNAi wings at 20.5 h APF at 29°C, which corresponds to ~24 h APF at 25°C. (b, c) Upper column: images obtained by maximum *z*-projection at the AJ plane. Lower column: images processed with a background-subtracted plug-in in ImageJ ($r = 100$), as carried out for other images of AIP1-GFP such as that shown in Fig. 1a. (b) An image of AIP1-GFP in cells outside the *ptc-Gal4*-expressing region (B region). Blue arrow points to the AP junction. (c) Images of AIP1-

GFP (left) and the AJ marker Dα-cat-TagRFP (right) in cells inside the *ptc-Gal4*-expressing region (C region). (d, e) Top-view (d) and side-view (e) of *tsr* RNAi cells expressing AIP1-GFP (left) and Dα-cat-TagRFP (right). The vertical section along the dashed arrow in (d) is shown in (e). Alphabets label the corresponding coordinates in different views.

(f-h) Images of a WT wing at 24 h APF. (f) Images of cofilin-GFP (left) and Dα-cat-TagRFP (right). (g, h) Top-view (g) and side-view (h) of cells expressing cofilin-GFP (left) and Dα-cat-TagRFP (right). The vertical section in along the dashed arrow in (g) is shown in (h). Alphabets label the corresponding coordinates in different views.

(i-k) Images of a *flr* RNAi wing at 24 h APF. (i) Images of cofilin-GFP (left) and Dα-cat-TagRFP (right). (j, k) Top-view (j) and side-view (k) of cells expressing cofilin-GFP (left) and Dα-cat-TagRFP (right). The vertical section along the dashed arrow in (j) is shown in (k). Alphabets label the corresponding coordinates in different views. Arrowheads indicate the accumulation of cofilin-GFP at the AJ plane.

Genotypes are *ptc-Gal4*, *tub-Gal80*^{ts}, *UAS-Dα-cat-TagRFP/UAS-tsr dsRNA*; *flr-GFP/flr-GFP* (b-e), *ptc-Gal4*, *UAS-Dα-cat-TagRFP/tsr-GFP* (f-h), and *ptc-Gal4*, *UAS-Dα-cat-TagRFP/tsr-GFP*, *UAS-flr dsRNA* (i-k).

Scale bars: 5 μm (b, c, f, i) and 2 μm (d, e, g, h, j, k).

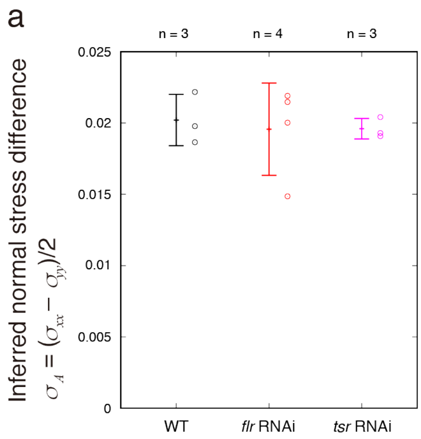

SUPPLEMENTARY FIGURE 4 The anisotropy of the tissue stress is not affected in the *flr* or *tsr* RNAi wings.

The force generated in the proximal region of the body stretches the wing along the PD axis, which builds up tensile tissue stress[S01,S02]. This tensile stress orients cell rearrangement toward the PD axis at 21-22 h APF and afterwards[S01-S04]. Using Bayesian force/stress inference[S02,S09], we assessed whether the anisotropy of tissue stress was affected in RNAi wings (see METHODS).

(a) The inferred normal stress difference $\sigma_A \equiv (\sigma_{xx} - \sigma_{yy})/2$, which is a measure for tissue stress anisotropy, for the genotype indicated. The *x*- and *y*-axes correspond to the PD and AP axes of the wing, respectively. σ_A was estimated from the first frames of the movies analyzed in Fig. 2. Data from each pupa (circle) and the mean ± s.d. are plotted. The number of pupae examined is indicated. Dunnett's test: WT vs. *flr* RNAi, $P > 0.9$, WT vs. *tsr* RNAi, $P > 0.9$.

Genotypes are *DE-cad-GFP* (a), *MS1096/X* or *Y*; *DE-cad-GFP*/*DE-cad-GFP*, *UAS-flr dsRNA* (a), and *ptc-Gal4*, *tub-Gal80^{ts}*, *DE-cad-GFP*/*DE-cad-GFP*, *UAS-tsr dsRNA* (a).

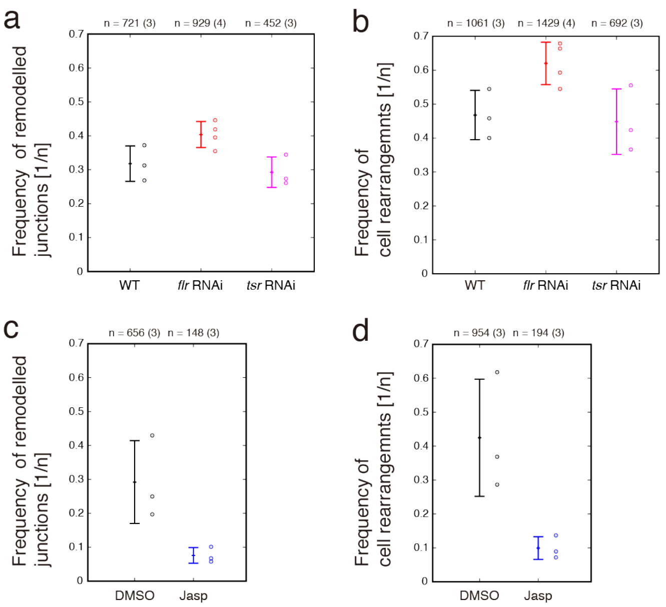

SUPPLEMENTARY FIGURE 5 Quantification of the frequency of cell rearrangement.
(a, b) Quantification of the frequency of cell rearrangement for each genotype from time-lapse data taken at 24–27 h APF at 25°C (WT, *flr* RNAi) and from time-lapse data obtained at 20.5 h–23 h 20 m APF at 29°C (*tsr* RNAi). The frequency of remodeled junctions (N_{seq}/N_{cell}) (a) and that of cell rearrangements (N_{all}/N_{cell}) (b) for each genotype are plotted (see Fig. 2e for their definition). Data from each pupa (circle) and the mean ± s.d. are plotted. The number of events and pupae examined are indicated. (a) Dunnett's test: WT vs. *flr* RNAi, P > 0.05, WT vs. *tsr* RNAi, P > 0.7. (b) Dunnett's test: WT vs. *flr* RNAi, P > 0.05, WT vs. *tsr* RNAi, P > 0.9.
(c, d) The frequency of cell rearrangement for each genotype from time-lapse data taken at 24–27 h APF at 25°C (DMSO-treated, Jasp-treated) was quantified as shown in (a, b). Welch's t-test: DMSO vs. Jasp, P > 0.05 (c). Welch's t-test: DMSO vs. Jasp, P > 0.05 (d).
Genotypes are *DE-cad-GFP* (a-d), *MS1096/X* or *Y*; *DE-cad-GFP/DE-cad-GFP*, *UAS-flr dsRNA* (a, b), and *ptc-Gal4*, *tub-Gal80^{ts}*, *DE-cad-GFP/DE-cad-GFP*, *UAS-tsr dsRNA* (a, b).

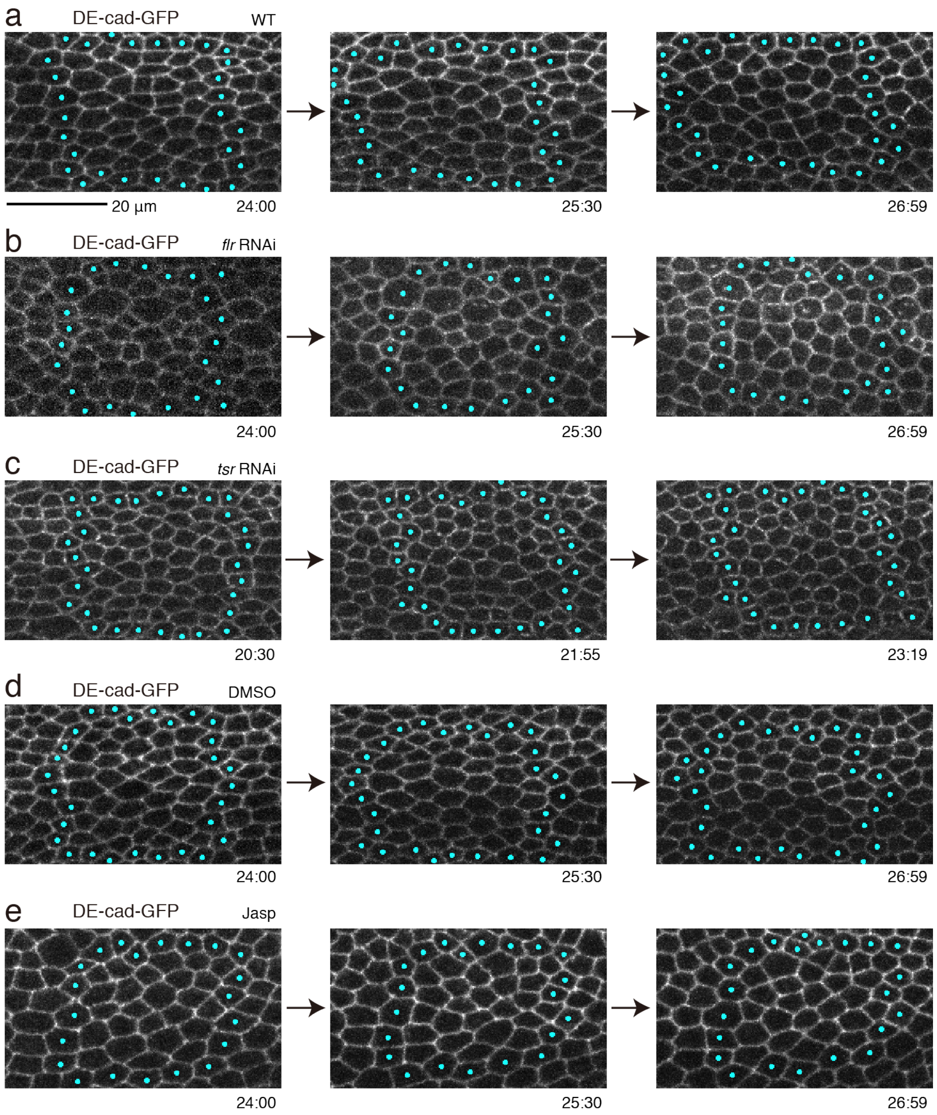

Supplementary Figure 6 Cell movements in genetically or pharmacologically manipulated wings.

(a-c) Still images from time-lapse recording of DE-cad-GFP (a, WT; b, *flr* RNAi; and c, *tsr* RNAi at 29°C). Blue dots label the same cells in consequent time frames. Wild type tissue shrinks along the AP axis and elongates along the PD axis (*i.e.*, contraction-elongation). The overall extent of contraction-elongation was decreased by *flr* or *tsr* RNAi.

(d, e) Cell movements in DMSO-treated (d) and Jasp-treated (e) wings are visualized as shown in (a-c).

Genotypes are *DE-cad-GFP* (a, d, e), *MS1096*/*X* or *Y*; *DE-cad-GFP*/*DE-cad-GFP*, *UAS-flr dsRNA* (b), and *ptc-Gal4*, *tub-Gal80*^{ts}, *DE-cad-GFP*/*DE-cad-GFP*, *UAS-tsr dsRNA* (c).

Scale bar: 20 µm (a).

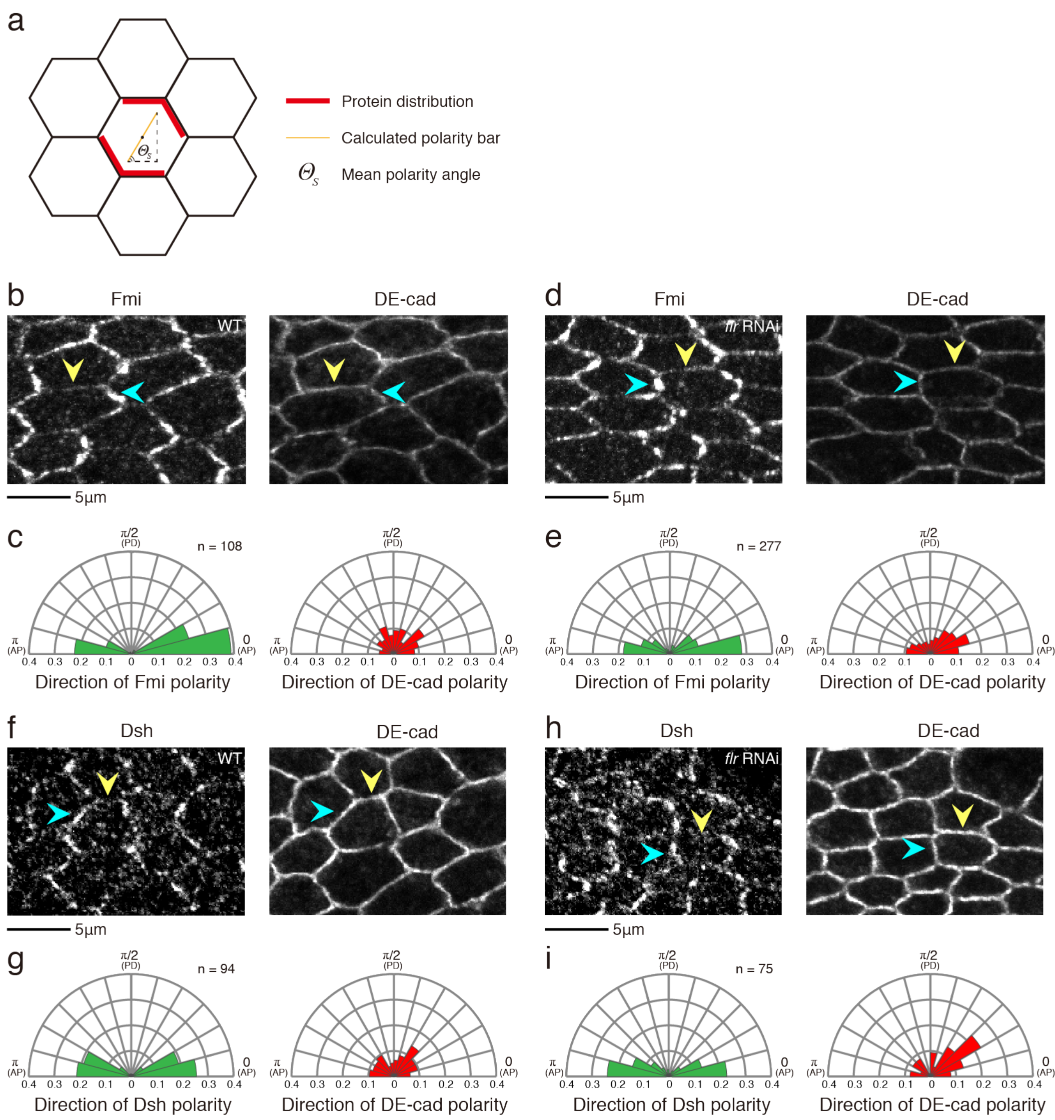

SUPPLEMENTARY FIGURE 7 Fmi and Dsh polarities are largely normal in the *flr* RNAi wing.

(a) Schematic of the quantification of PCP nematics[S01]. Briefly, the signal intensities of proteins (red) and the angles relative to the cell center were measured for each pixel in an ROI, from which the angle of polarity bar (light orange) (Θs) was calculated. See METHODS for details.

(b, d) WT (b) and *flr* RNAi wings (d) at 24 hr APF were stained for Fmi (left in b, d) and DE-cad (right in b, d). Yellow and blue arrowheads indicate PD and AP junctions, respectively.

(c, e) The polarity of protein distribution was as depicted in (a). The distribution of polarity angles in each cell was plotted in rose diagrams (WT, Fmi, left in c; WT, DE-cad, right in c; *flr* RNAi, Fmi, left in e; *flr* RNAi, DE-cad, right in e). 0 and π correspond to the biased distribution along the vertical, AP junctions, and $\pi/2$ corresponds to the biased distribution along the horizontal, PD junctions. Randomized version of Watson's test: WT (Fmi) vs. *flr* RNAi (Fmi), $P < 0.001$.

(f, h) WT (f) and *flr* RNAi wings (h) at 24 h APF were stained for Dsh (left in f, h) and DE-cad (right in f, h). Yellow and blue arrowheads indicate PD and AP junctions, respectively.

(g, i) The distribution of the polarity angles in each cell was plotted in rose diagrams (WT, Dsh, left in g; WT, DE-cad, right in g; *flr* RNAi, Dsh, left in i; *flr* RNAi, DE-cad, right in i). Randomized version of Watson's test: WT (Dsh) vs. *flr* RNAi (Dsh), $P > 0.4$.

The number of cells examined (c, e, g, i) is indicated.

Genotypes are *DE-cad-GFP* (b, c, f, g), and *MS1096/X* or *Y*; *DE-cad-GFP/ DE-cad-GFP*, *UAS-flr dsRNA* (d, e, h, i).

Scale bars: 5 µm (b, d, f, h).

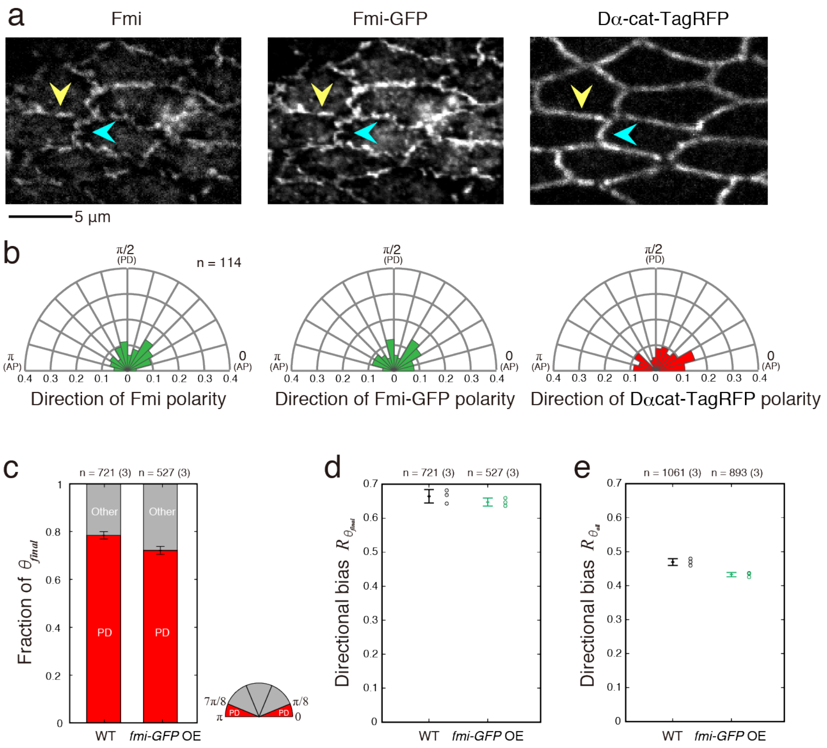

SUPPLEMENTARY FIGURE 8 The directionality of cell rearrangement was only mildly affected in *fmi-GFP* overexpression (OE) cells compared with *flr* RNAi cells.

(a) The *fmi-GFP* overexpressing wings at 20.5 h APF at 29°C, which corresponds to ~24 h APF at 25°C were stained for anti-Fmi. Images of Fmi (left), Fmi-GFP (middle), and Dα-cat-TagRFP (right) are shown. Yellow and blue arrowheads indicate PD and AP junctions, respectively.

(b) The polarity of protein distribution in the *fmi-GFP* overexpressing cells was quantified as shown in Supplementary Figure 7a (Fmi, left; Fmi-GFP, middle; Dα-cat-TagRFP, right). Randomized version of Watson's test: WT (Fmi) vs. *fmi-GFP* OE (Fmi), $P < 0.001$.

(c-e) Cell rearrangement defects induced by *fmi-GFP* overexpression were quantified as shown in Fig. 2e-h. (c) PD (red) and other (gray) fractions of θ_{final}. (d, e) $R_{\theta final}$ (d) and $R_{\theta all}$ (e) for each condition. The directionality of cell rearrangement was only mildly affected compared with *flr* RNAi cells. (d) Welch's t-test: WT vs. *fmi-GFP* OE, $P > 0.2$. (e) Welch's t-test: WT vs. *fmi-GFP* OE, $P < 0.01$.

The number of cells (b) and the numbers of events and pupae examined (c-e) are indicated. Data are presented as the mean ± s.d. (d, e).

Genotypes are *ptc-Gal4*, *tub-Gal80*^{ts}, *UAS-Dα-cat-TagRFP*/+; *UAS-fmiTTV:3eGFP*/+ (a-e), and *DE-cad-GFP* (c-e).

Scale bar: 5 µm (a).

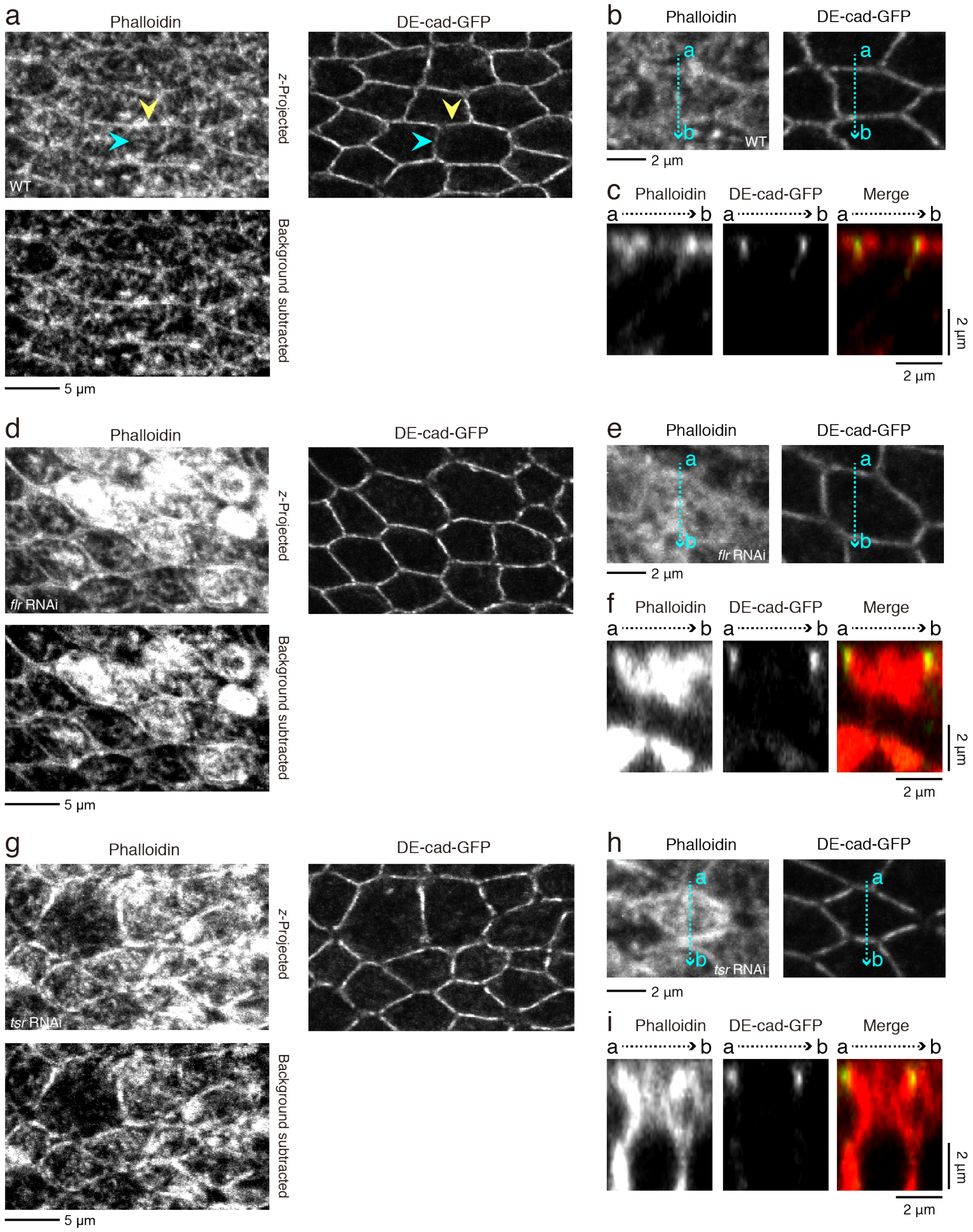

Supplementary Figure 9 *flr* or *tsr* RNAi resulted in the abnormal accumulation of F-actin. (a-i) F-actin distribution in wings of the indicated genotypes (a-c, WT at 24 h APF; d-f, *flr* RNAi at 24 h APF; and g-i, *tsr* RNAi at 20.5 h APF at 29°C respectively, which corresponds to ~24 h APF at 25°C). Phalloidin (gray in left panels in a-i, red in right panels in c, f, i) and DE-cad-GFP (gray in right panels in a, b, d, e, g, h, gray in middle panels in c, f, i, red in right panels in c, f, i). (a, d, g) Top-view of the AJ plane. Images in (a) are the same as those shown in Fig. 3e. Upper column: images obtained by maximum *z*-projection at the AJ plane. Lower column: images processed with a background-subtracted plug-in in ImageJ (r = 100). (b, c, e, f, h, i) Top-view (b, e, h) and side-view (c, f, i) of wings of the indicated genotypes. The vertical sections (c, f, i) are constructed along the dashed arrows in (b, e, h) by using ImageJ. Alphabets indicate corresponding coordinates in different views.

Genotypes are *DE-cad-GFP* (a-c), *MS1096/X* or *Y*; *DE-cad-GFP/DE-cad-GFP*, *UAS-flr dsRNA* (d-f), and *ptc-Gal4*, *tub-Gal80^{ts}*, *DE-cad-GFP/DE-cad-GFP*, *UAS-tsr dsRNA* (g-i).
Scale bars: 5 µm (a, d, g) and 2 µm (b, c, e, f, h, i).

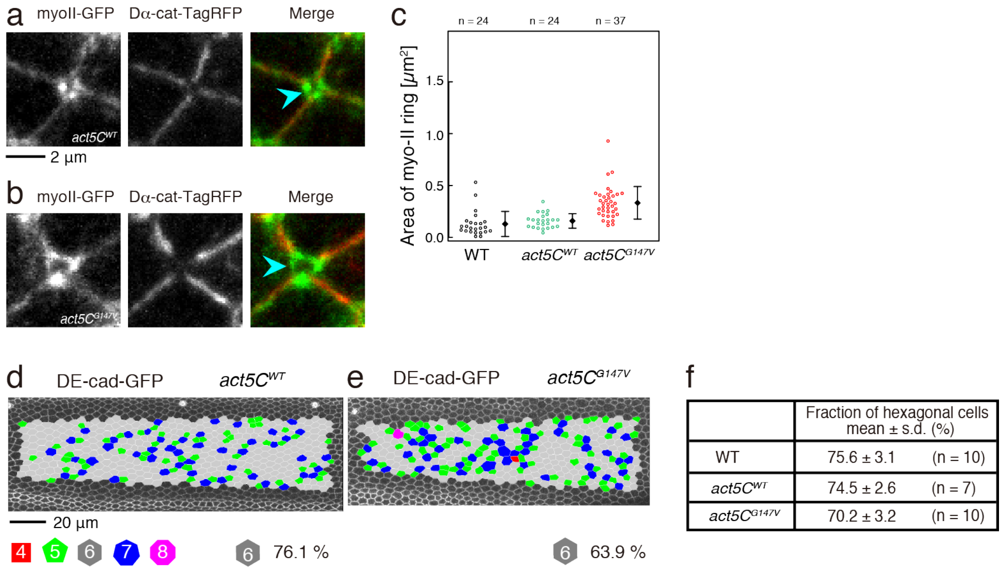

SUPPLEMENTARY FIGURE 10 Misexpression of mutant *actin5C* but not its wildtype counterpart induced the detachment of junctional actomyosin and the decrease of hexagonal cells.

(a, b) Images of myo-II-GFP (gray in left panels, green in right panels) and Dα-cat-TagRFP (gray in middle panels, red in in right panels) in *act5C*^{WT} misexpressed (a) and *act5C*^{G147V} misexpressed (b) wings at 24 h APF at 25°C. Blue arrowhead indicates myo-II ring.

(c) Myo-II ring area. Steel-Dwass test: WT vs. *act5C*^{WT}, $P < 0.05$, WT vs. *act5C*^{G147V}, $P < 0.001$, *act5C*^{WT} vs. *act5C*^{G147V}, $P < 0.001$.

(d, e) Images of DE-cad-GFP with the indicated genotypes at 32 h APF at 25°C (d, *act5C*^{WT} misexpression; e, *act5C*^{G147V} misexpression). Cells are colored according to the number of junctions.

(f) Table listing the fractions of hexagonal cells for each genotype. Steel-Dwass test: WT vs. *act5C*^{WT}, $P > 0.6$, WT vs. *act5C*^{G147V}, $P < 0.05$, *act5C*^{WT} vs. *act5C*^{G147V}, $P < 0.05$.

The number of ROIs examined (c), and the number of pupae examined (f) are indicated. Data are presented as the mean ± s.d. (c, f).

Genotypes are *MS1096/X* or *Y*; *UAS-Dα-cat-TagRFP*, *sqhp-sqh-GFP/UAS-attB-act5C*^{WT}; *UAS-attB-act5C*^{WT}*/+* (a, c), *MS1096/X* or *Y*; *UAS-Dα-cat-TagRFP*, *sqhp-sqh-GFP/UAS-attB-act5C*^{G147V}; *UAS-attB-act5C*^{G147V}*/+* (b, c), *MS1096/X* or *Y*; *DE-cad-GFP/UAS-attB-act5C*^{WT}; *UAS-attB-act5C*^{WT}*/+* (d, f), *MS1096/X* or *Y*; *DE-cad-GFP/UAS-attB-act5C*^{G147V}; *UAS-attB-act5C*^{G147V}*/+* (e, f), and *DE-cad-GFP* (f).

Scale bars: 2 μm (a) and 20 μm (d).

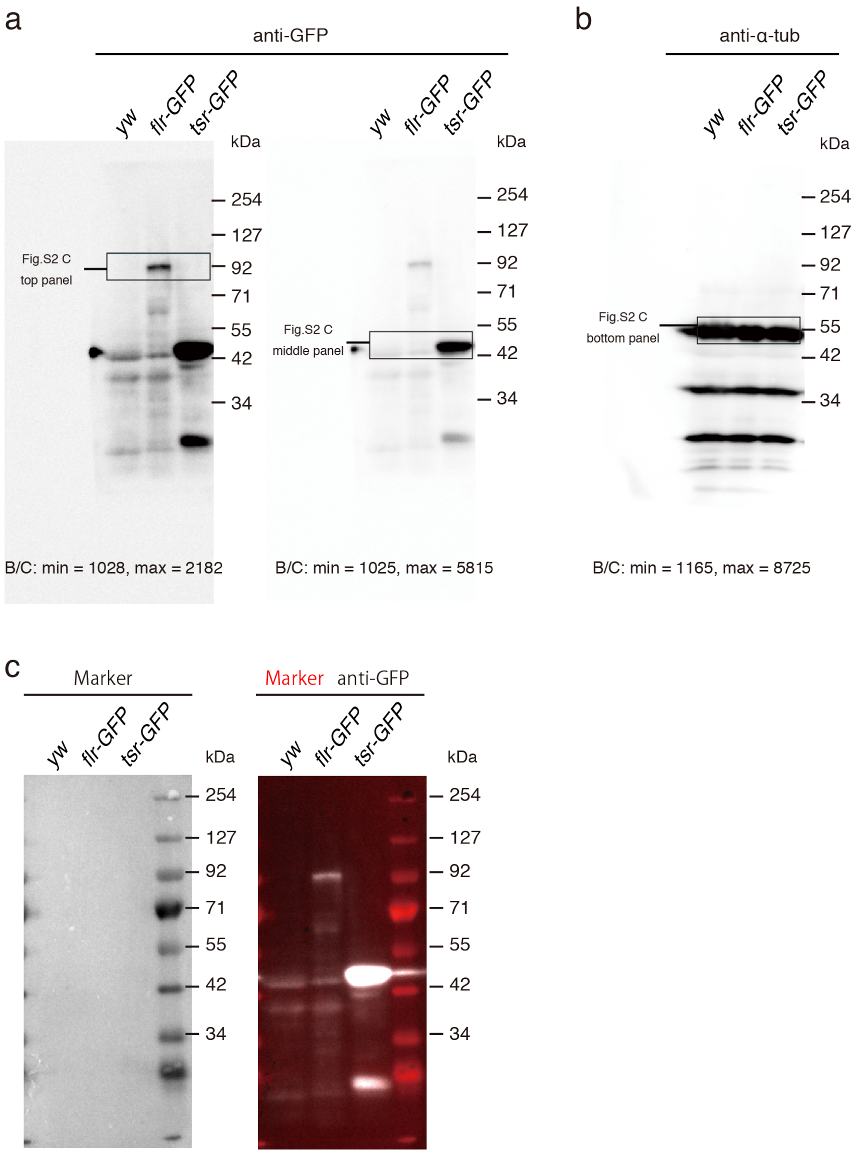

Supplementary Figure 11 Original scan images of Supplementary Figure 2c.
(a, b) Immunoblotting using anti-GFP antibody (a) and anti-α-tubulin antibody (b). Genotypes of the pupae are indicated at the top. Rectangular regions were cropped and shown in Supplementary Figure 2c. Horizontal lines indicate the molecular marker. Parameters for brightness-contrast adjustment are indicated below. B/C indicates the brightness and contrast. In (a), different parameters for brightness-contrast adjustment were used to visualize AIP1-GFP (left) and cofilin-GFP (right). (b) α-tubulin was used as a loading control.
(c) The anti-GFP immunoblotting image shown in (a) (gray in a right panel) is overlaid with an image of a membrane (gray in a left panel and red in a right panel) to indicate the positions of the molecular weight markers indicated on the right.
Genotypes are *yw*, *flr-GFP*, and *tsr-GFP* (a-c).

SUPPLEMENTARY NOTES

Supplementary Note 1 Characterization of *flr* and *tsr* protein trap lines

In the *flare-GFP* (#CA07499) line[S10], an artificial exon encoding GFP is inserted at 3L: 13413131 in version FB2017_06 (http://flybase.org/reports/FBal0211615.html), which lies within the second intron of the *flr* gene, thus, GFP is inserted after Asn-11 of Flr-PA (Supplementary Figure 2a). The AIP1-GFP fusion protein was detected using western blotting at the expected size as has been reported previously (Supplementary Figure 2c)[S06]. It has been shown that immunostaining using the anti-AIP1 antibody and fluorescent imaging using AIP1-GFP constructs showed an identical pattern in the eye disc[S06]. The *flr-GFP* line is homozygous viable, and the eye[S06] and wing develop normally. These data verify the validity of the *flare-GFP* line as a tool to monitor the distribution of AIP1.

We determined an insertion site for an artificial exon encoding GFP in *tsr-GFP* (#ZCL0613)[S11] as 2R: 24045791 in version FB2017_06, which lies within the first intron of the *tsr* gene and upstream of the first codon (Supplementary Figure 2b; Methods). The cofilin-GFP fusion protein of the expected size was detected by western blotting (Supplementary Figure 2c). The *tsr-GFP* line is homozygous-viable and exhibited no discernible defects in the adult wing, suggesting that the GFP-cofilin protein is functional.

Supplementary Note 2 The strength of *flr* and *tsr* RNAi phenotypes

The strength of *flr* or *tsr* RNAi phenotypes depended on the timing of the temperature shift. Therefore, we carefully adjusted this timing to bypass early developmental defects and avoid inhibiting junction dynamics in the C region of the wing. For example, when temperature was shifted up 25 hours earlier, cell shape was severely deformed and cell division was partially inhibited (compare Supplementary Figure. 2h and i; see Supplementary Figure 2m and n for *tsr* RNAi experiments). *flr* or *tsr* RNAi flies were raised as in Supplementary Figure 2h, m throughout this study. The GFP-signal of AIP1-GFP or Cofilin-GFP was not detected under experimental conditions employed (Supplementary Figure 2d-f, j). Compared with the drastic increase in phalloidin intensity and the malformation of adult wing hairs in *flr* or *tsr* null mutant clone cells[S12], *flr* RNAi and *tsr* RNAi phenotypes were slightly weaker (Supplementary Figure 2g, k, l; Supplementary Figure 9), indicating that *flr* RNAi and *tsr* RNAi are strong downregulations, but not null conditions. The difference in the strength of the l-o-f conditions may explain the discrepancy with previous studies: why Fmi and Dsh polarities were largely normal in the *flr* RNAi wing (Supplementary Figure 7), unlike in the *flr* null mutant clone cells[S12].

SUPPLEMENTARY METHODS

Screening of actin binding proteins (ABPs)

Based on a literature search[S13,S14], we identified 19 ABPs for investigation. We observed the subcellular localization of each ABP at 24 h APF using the reagents listed below.

ABP	Reagents	Provider
AIP1	*flr-GFP* (Flytrap)[S10]	Flytrap
Arp3	anti-Arp3[S15]	Tatsuhiko Noguchi
cofilin	*tsr-GFP* (Flytrap)[S11],<br>anti-cofilin[S16], and anti-phospho-cofilin[S16]	Flytrap<br>Tadashi Uemura<br>Tadashi Uemura
Coronin	*UAS-coronin-EGFP*	This study
Coronin-7	*UAS-pod1.GFPmyc*[S17]	Bloomington #8800
Diaphanous	*UAS-DiaGFP*[S18]	Steven A. Wasserman
Enabled	*Ubi-GFP.ena*[S19]	Bloomington #28798
Fascin	*UAS-mKO-sn*[S20]	Gaia Tavosanis
Filamin A	anti-Cher[S21]	Mirka Uhlirova
Lasp	*Lasp-GFP* (Flytrap)[S22]	Flytrap
LIM kinase-1	*UAS-LIMK1-HA*[S16]	Tadashi Uemura
Myosin heavy chain	anti-Zip[S23]	Fumio Matsuzaki
Myosin regulatory light chain	*sqhp-sqh-GFP*[S24]	Roger Karess
Profilin	anti-Profilin[S25]	DSHB chi 1J
SCAR	anti-SCAR[S26]	DSHB P1C1-SCAR
Slingshot	anti-Ssh[S16]	Tadashi Uemura
Twinfilin	anti-Twf[S27]	Tapio I. Heino
WASp	*sqhp-WASp-RBD-GFP*[S28]	Bloomington #56745, #56746
Zyxin	*UAS-Zyx-ChRFP*	Bloomington #28875

Supplementary Table 1 Reagents used in the ABP screening.

Drosophila genetics

The flies used in the present study were *flr-GFP* (Flytrap #CA07499)[S10], *tsr-GFP* (Flytrap #ZCL0613)[S11], *UAS-Dα-cat-TagRFP*[S09], *DE-cad-GFP*[S29], *Ubi-GFP.ena*[S19], *sqhp-sqh-GFP*[S24], *sqhp-utrABD-GFP*[S30], *ptc-Gal4*, *MS1096-Gal4*, *tubP-gal80*^{ts} (Bloomington Stock Center #7108), *UAS-flr dsRNA* (VDRC #v108422), *UAS-tsr dsRNA* (VDRC #v110599), *pk*^{1} (ref. S31), *UAS-fmi dsRNA*[S32], *UAS-fmiTTV:3eGFP*[S33], *UAS-cno dsRNA* (NIG-Fly #2534R-2), *UAS-act5C*^{G147V}, *UAS-attB-act5C*^{WT}, and *UAS-attB-act5C*^{G147V} (this study). Fly genotypes and culture conditions are summarized below.

Genotype	Culture condition	Figure
ptc-Gal4, *UAS-Dα-cat-TagRFP*/+; *flr-GFP*	White pupae were picked up and observed at 25°C.	Figure 1, 4 Supplementary Figure 1 Supplementary Movie 1, 2
DE-cad-GFP	White pupae were picked up and observed at 25°C.	Figure 2-4, 9, Supplementary Figure 1, 4-7, 9, 10
MS1096/*X* or *Y*; *DE-cad-GFP*/ *DE-cad-GFP*, *UAS-flr dsRNA*	Crossed at 17°C. White pupae were picked up and observed at 25°C.	Figure 2, Supplementary Figure 2, 4-7, 9
MS1096/*X* or *Y*; *DE-cad-GFP*/ *DE-cad-GFP*, *UAS-flr dsRNA*	Crossed at 17°C. During the 3rd instar stage, temperature was shifted up at 25°C. After 25 h, white pupae were picked up and observed at 25°C.	Supplementary Figure 2
ptc-Gal4, *tub-Gal80^{ts}*, *DE-cad-GFP*/ *DE-cad-GFP*, *UAS-tsr dsRNA*	Crossed at 21°C. During the 3rd instar stage, temperature was shifted up at 29°C. After 20-22 h, white pupae were picked up and observed at 29°C.	Figure 2, Supplementary Figure 2, 4-6, 9
ptc-Gal4, *tub-Gal80^{ts}*, *DE-cad-GFP*/ *DE-cad-GFP*, *UAS-tsr dsRNA*	Crossed at 21°C. During the 3rd instar stage, temperature was shifted up at 29°C. After 25 h, white pupae were picked up and observed at 29°C.	Supplementary Figure 2
Ubi-ena-GFP	White pupae were picked up and observed at 25°C.	Figure 3
pk^{1}; *flr-GFP*	White pupae were picked up and observed at 25°C.	Figure 3
ptc-Gal4, UAS-Dα-cat-TagRFP/+; *UAS-fmi dsRNA*/*flr-GFP*	Crossed and observed at 25°C.	Figure 3
MS1096/*X* or *Y*; *UAS-Dα-cat-TagRFP*/*sqhp-sqh-GFP*	Crossed at 17°C. White pupae were picked up and observed at 25°C.	Figure 5-9
MS1096/*X* or *Y*; *UAS-Dα-cat-TagRFP*/*sqhp-sqh-GFP*, *UAS-flr dsRNA*	Crossed at 17°C. White pupae were picked up and observed at 25°C.	Figure 5-7
ptc-Gal4, *tub-Gal80^{ts}*, *UAS-Dα-cat-TagRFP*/*sqh-GFP*, *UAS-tsr dsRNA*	Crossed at 21°C. During the 3rd instar stage, temperature was shifted up at 29°C. After 21-23 h, white pupae were picked up and observed at 29°C.	Figure 6
MS1096/*X* or *Y*; *UAS-Dα-cat-TagRFP*, *sqhp-sqh-GFP*/+; *UAS-cno dsRNA*/+	Crossed and observed at 25°C.	Figure 6
ptc-Gal4, *sqhp-utrABD-GFP*/+	Crossed at 17°C. White pupae were picked up and observed at 25°C.	Figure 1, 8
ptc-Gal4, *sqhp-utrABD-GFP*/*UAS-flr dsRNA*	Crossed at 17°C. White pupae were picked up and observed at 25°C.	Figure 8
ptc-Gal4, *tub-Gal80^{ts}*, *UAS-Dα-cat-TagRFP*/*UAS-tsr dsRNA*, *sqhp-utrABD-GFP*	Crossed at 21°C. During the 3rd instar stage, temperature was shifted up at 29°C. After 20-23 h, white pupae were picked up and observed at 29°C.	Figure 8
MS1096/*X* or *Y*; *UAS-Dα-cat-TagRFP*/+; *flr-GFP*, *UAS-act5C^{G147V}*/+	Crossed and observed at 29°C.	Figure 9

MS1096/X or *Y*; *UAS-Dα-cat-TagRFP*, *sqhp-sqh-GFP*/*UAS-act5C*^{G147V}, *UAS-act5C*^{G147V}	Crossed and observed at 29°C.	Figure 9
MS1096/X or *Y*; *DE-cad-GFP*/*UAS-act5C*^{G147V}, *UAS-act5C*^{G147V}	Crossed and observed at 29°C.	Figure 9
yw	Flies were raised at 25°C.	Supplementary Figure 2, 11
flr-GFP	Flies were raised at 25°C.	Supplementary Figure 2, 11
tsr-GFP	Flies were raised at 25°C.	Supplementary Figure 2, 11
MS1096; *UAS-Dα-catenin-TagRFP*/+; *flr-GFP*/+	Crossed at 17°C. White pupae were picked up and observed at 25°C.	Supplementary Figure 2
MS1096; *UAS-Dα-catenin-TagRFP*/*UAS-flare dsRNA*; *flr-GFP*/+	Crossed at 17°C. White pupae were picked up and observed at 25°C.	Supplementary Figure 2
ptc-Gal4, tub-Gal80^{ts}*, UAS-Dα-catenin-TagRFP/tsr-GFP, UAS-tsr dsRNA*	Crossed at 21°C. During the 3rd instar stage, temperature was shifted up at 29°C. After 20-22 h, white pupae were picked up and observed at 29°C.	Supplementary Figure 2
ptc-Gal4, *tub-Gal80*^{ts}, *UAS-Dα-cat-TagRFP*/*UAS-tsr dsRNA*; *flr-GFP*/*flr-GFP*	Crossed at 21°C. During the 3rd instar stage, temperature was shifted up at 29°C. After 20-22 h, white pupae were picked up and observed at 29°C.	Supplementary Figure 3
ptc-Gal4, *UAS-Dα-cat-TagRFP*/*tsr-GFP*	Crossed at 17°C. White pupae were picked up and observed at 25°C.	Supplementary Figure 3
ptc-Gal4, *UAS-Dα-cat-TagRFP*/ *tsr-GFP*, *UAS-flr dsRNA*	Crossed at 17°C. White pupae were picked up and observed at 25°C.	Supplementary Figure 3
ptc-Gal4, *tub-Gal80*^{ts}, *UAS-Dα-cat-TagRFP*/+; *UAS-fmiTTV:3eGFP*/+	Crossed at 21°C. White pupae were picked up and observed at 29°C.	Supplementary Figure 8
MS1096/X or *Y*; *UAS-Dα-cat-TagRFP*, *sqhp-sqh-GFP*/*UAS-attB-act5C*^{WT}; *UAS-attB-act5C*^{WT}/+	Crossed and observed at 25°C.	Supplementary Figure 10
MS1096/X or *Y*; *UAS-Dα-cat-TagRFP*, *sqhp-sqh-GFP*/*UAS-attB-act5C*^{G147V}; *UAS-attB-act5C*^{G147V}/+	Crossed and observed at 25°C.	Supplementary Figure 10
MS1096/X or *Y*; *DE-cad-GFP*/ *UAS-attB-act5C*^{WT}; *UAS-attB-act5C*^{WT}/+	Crossed and observed at 25°C.	Supplementary Figure 10
MS1096/X or *Y*; *DE-cad-GFP*/ *UAS-attB-act5C*^{G147V}; *UAS-attB-act5C*^{G147V}/+	Crossed and observed at 25°C.	Supplementary Figure 10

Supplementary Table 2 Fly genotypes and culture conditions.

SUPPLEMENTARY REFERENCES

S01. Aigouy, B. *et al.* Cell flow reorients the axis of planar polarity in the wing epithelium of *Drosophila*. *Cell* **142**, 773-786 (2010).

S02. Sugimura, K. & Ishihara, S. The mechanical anisotropy in a tissue promotes ordering in hexagonal cell packing. *Development* **140**, 4091-4101 (2013).

S03. Etournay, R. *et al.* Interplay of cell dynamics and epithelial tension during morphogenesis of the *Drosophila* pupal wing. *eLife* **4**, e07090 (2015).

S04. Matamoro-Vidal, A., Salazar-Ciudad, I. & Houle, D. Making quantitative morphological variation from basic developmental processes: Where are we? The case of the *Drosophila* wing. *Dev Dyn* **244**, 1058-1073 (2015).

S05. Guirao, B. *et al.* Unified quantitative characterization of epithelial tissue development. *eLife* **4**, e08519 (2015).

S06. Chu, D. *et al.* AIP1 acts with cofilin to control actin dynamics during epithelial morphogenesis. *Development* **139**, 3561-3571 (2012).

S07. Okada, K., Ravi, H., Smith, E.M. & Goode, B.L. Aip1 and cofilin promote rapid turnover of yeast actin patches and cables: a coordinated mechanism for severing and capping filaments. *Mol Biol Cell* **17**, 2855-2868 (2006).

S08. Lin, M.C., Galletta, B.J., Sept, D. & Cooper, J.A. Overlapping and distinct functions for cofilin, coronin and Aip1 in actin dynamics *in vivo*. *J Cell Sci* **123**, 1329-1342 (2010).

S09. Ishihara, S. & Sugimura, K. Bayesian inference of force dynamics during morphogenesis. *J Theor Biol* **313**, 201-211 (2012).

S10. Buszczak, M. *et al.* The Carnegie protein trap library: A versatile tool for *Drosophila* developmental studies. *Genetics* **175**, 1505-1531 (2007).

S11. Morin X., Daneman R., Zavortink M., & China W. A protein trap strategy to detect GFP-tagged proteins expressed from their endogenous loci in *Drosophila*. *Proc Natl Acad Sci U S A* **98**, 15050-15055 (2001).

S12. Ren, N., Charlton, J. & Adler, P.N. The *flare* gene, which encodes the AIP1 protein of *Drosophila*, functions to regulate F-actin disassembly in pupal epidermal cells. *Genetics* **176**, 2223-2234 (2007).

S13. Rogers, L.S., Wiedemann, U., Stuurman, N., Vale D.R. Molecular requirments for actin-based lamella formation in *Drosophila* S2 cells. *J Cell Biol* **162**, 1079-1088 (2003).

S14. Rohn L.J *et al.* Cmparative RNAi screening identifies a conserved core metazoan actinome by phenotype. *J Cell Biol* **194**, 789-805 (2011).

S15. Stevenson, V., Hudson, A., Cooley, L. & Theurkauf, W.E. Arp2/3-dependent pseudocleavage furrow assembly in syncytial *Drosophila* embryos. *Curr Biol* **12**, 705-711 (2002).

S16. Niwa, R., Nagata-Ohashi, K., Takeichi, M., Mizuno, K. & Uemura, T. Control of actin reorganization by Slingshot, a family of phosphatases that dephosphorylate ADF/cofilin. *Cell* **108**, 233-246 (2002).

S17. Rothenberg, M.E., Rogers, S.L., Vale, R.D., Jan, L.Y. & Jan, Y.N. *Drosophila* pod-1 crosslinks both actin and microtubules and controls the targeting of axons. *Neuron* **39**, 779-791 (2003).

S18. Afshar, K., Stuart, B. & Wasserman, S.A. Functional analysis of the *Drosophila* diaphanous FH protein in early embryonic development. *Development* **127**, 1887-1897 (2000).

S19. Bilancia, C.G. *et al.* Enabled negatively regulates diaphanous-driven actin dynamics *in vivo* and *in vivo*. *Dev Cell* **28**, 394-408 (2014).

S20. Nagel, J. *et al.* Fascin controls neuronal class-specific dendrite arbor morphology. *Development* **139**, 2999-3009 (2012).

S21. Sokol, N.S. & Cooley, L. *Drosophila* filamin encoded by the cheerio locus is a component of ovarian ring canals. *Curr Biol* **9**, 1221-1230 (1999).

S22. Quinones-Coello, A.T. *et al.* Exploring strategies for protein trapping in *Drosophila*. *Genetics* **175**, 1089-1104 (2007).

S23. Sano, H. *et al.* The *Drosophila* actin regulator ENABLED regulates cell shape and orientation during gonad morphogenesis. *PLoS One* **7**, e52649 (2012).

S24. Royou, A., Field, C., Sisson, J.C., Sullivan, W. & Karess, R. Reassessing the role and dynamics of nonmuscle myosin II during furrow formation in early *Drosophila* embryos. *Mol Biol Cell* **15**, 838-850 (2004).

S25. Verheyen, E.M. & Cooley, L. Profilin mutations disrupt multiple actin-dependent processes during *Drosophila* development. *Development* **120**, 717-728 (1994).

S26. Rodriguez-Mesa, E., Abreu-Blanco, M.T., Rosales-Nieves, A.E. & Parkhurst, S.M. Developmental expression of *Drosophila* Wiskott-Aldrich Syndrome family proteins. *Dev Dyn* **241**, 608-626 (2012).

S27. Wahlstrom, G. *et al.* Twinfilin is required for actin-dependent developmental processes in *Drosophila*. *J Cell Biol* **155**, 787-796 (2001).

S28. Abreu-Blanco, M.T., Verboon, J.M. & Parkhurst, S.M. Coordination of Rho family GTPase activities to orchestrate cytoskeleton responses during cell wound repair. *Curr Biol* **24**, 144-155 (2014).

S29. Huang, J., Zhou, W., Dong, W., Watson, A.M. & Hong, Y. From the Cover: Directed, efficient, and versatile modifications of the *Drosophila* genome by genomic engineering. *Proc Natl Acad Sci U S A* **106**, 8284-8289 (2009).

S30. Rauzi, M., Lenne, P.F. & Lecuit, T. Planar polarized actomyosin contractile flows control epithelial junction remodeling. *Nature* **468**, 1110-1114 (2010).

S31. Jenny, A., Reynolds-Kenneally, J., Das, G., Burnett, M. & Mlodzik, M. Diego and Prickle regulate Frizzled planar cell polarity remodeling by competing for Dishevelled binding. *Nat Cell Biol* **7**, 691-697 (2005).

S32. Shimada, Y., Yonemura, S., Ohkura, H., Strutt, D. & Uemura, T. Polarized transport of Frizzled along the planar microtubule arrays in *Drosophila* wing epithelium. *Dev Cell* **10**, 209-222 (2006).

S33. Matsubara, D., Horiuchi, S.Y., Shimono, K., Usui, T. & Uemura, T. The seven-pass transmembrane cadherin Flamingo controls dendritic self-avoidance via its binding to a LIM domain protein, Espinas, in *Drosophila* sensory neurons. *Genes Dev* **25**, 1982-1996 (2011).
