# Supplementary material for: AIP1 and cofilin ensure a resistance to tissue tension and promote directional cell rearrangement
Source: Nat Commun. 2018 Sep 10;9:3295. doi: 10.1038/s41467-018-05605-7 (PMC6131156; doi:10.1038/s41467-018-05605-7)
Supplement: Supplementary file 2 — Description of Additional Supplementary Files [file 41467_2018_5605_MOESM2_ESM.pdf]

Description of Additional Supplementary Files

File Name: Supplementary Movie 1
Description: Temporal change in the AIP1-GFP signal intensity during PD cell rearrangement. Time-lapse recording of AIP1-GFP (top, green in bottom) and Dα-cat-TagRFP (red in bottom) in a WT wing. Snapshots are shown in Fig. 1h. Scale bar: 5 µm

File Name: Supplementary Movie 2
Description: Temporal change in the AIP1-GFP signal intensity along non-remodeling junction. Time-lapse recording of AIP1-GFP (top, green in bottom) and Dα-cat-TagRFP (red in bottom) in a WT wing. Snapshots are shown in Fig. 1j. Scale bar: 5 µm
